# All-van der Waals microcavities for low-loss nonlinear photonics

**DOI:** 10.1038/s41563-026-02574-x

**Published:** 2026-04-13

**Authors:** Zhi-Yan Wang, Xiaoqi Cui, Andreas C. Liapis, Hao-Ran Shao, Xu Cheng, Jingnan Yang, Nianze Shang, Weizhe Zhang, Henri Kaaripuro, Juan C. Arias Muñoz, Kaifeng Lin, Wenjing Liu, Kaihui Liu, Qihuang Gong, Zhipei Sun, Yun-Feng Xiao

**Affiliations:** 1 State Key Laboratory for Mesoscopic Physics and Frontiers Science Centre for Nano-optoelectronics, School of Physics, Peking University, Beijing, China; 2 Department of Electronics and Nanoengineering, Aalto University, Espoo, Finland; 3 Collaborative Innovation Centre of Extreme Optics, Shanxi University, Taiyuan, China; 4 New Cornerstone Science Laboratory, School of Physics, Peking University, Beijing, China

**Keywords:** Nonlinear optics, Two-dimensional materials

## Abstract

Van der Waals (vdW) materials have emerged as a promising platform for next-generation nanophotonics and optoelectronics. However, employing vdW materials as a core photonic integration platform, rather than as passive or active overlays on conventional silicon-based platforms, remains challenging, leaving their full potential untapped. Here we develop a nanofabrication strategy that enables high-resolution patterning across a broad range of vdW materials, including insulators, semiconductors, ferroelectrics and their heterostructures, and we show that they can be used as the intrinsic platform for low-loss microcavity nonlinear photonic devices such as microdisks, photonic crystals and metasurfaces. We demonstrate vdW microdisk resonators with quality (*Q*) factors exceeding 10^6^. Such *Q* factors enable efficient continuous-wave nonlinear optical processes, including second-harmonic generation, sum-frequency generation and optical parametric amplification, with full free-spectral-range thermal tunability. These results position vdW materials as key material building blocks for next-generation integrated photonics and optoelectronics.

## Abstract (web summary)

Photonic components fabricated from van der Waals materials and patterned using focused ion beam lithography with aluminium passivation demonstrate quality factors exceeding 10^6^ and an efficient nonlinear optical response.

## Main

**Fig. 1:**
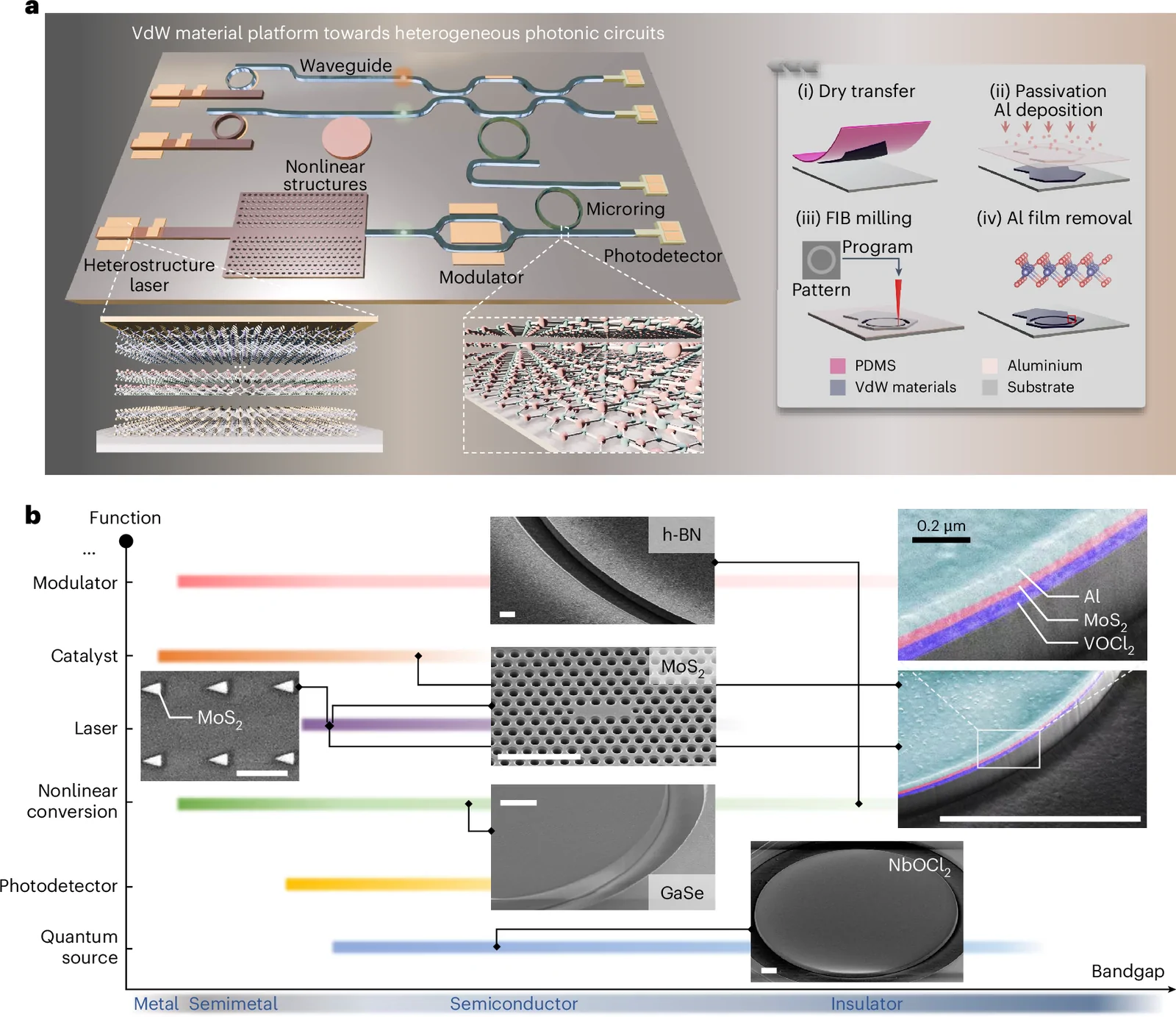
All-vdW microcavity photonics. **a**, Visionary conceptual illustration of a heterogeneous photonic chip based on vdW materials. It demonstrates the integration of exclusively vdW materials through techniques such as transferring, stacking and patterning, which enables the fabrication of lasers, modulators, waveguides, photodetectors and so on. Inset: fabrication workflow based on FIB milling with Al passivation. **b**, Various nanostructures fabricated from representative vdW materials (for example, h-BN, MoS_2_, GaSe and NbOCl_2_), including microdisk cavities, arrays of triangular microresonators and photonic crystal cavities. The structures span materials with different bandgaps and include heterostructure examples (for example, a VOCl_2_/MoS_2_ stack). Scale bars, 2.5 μm.

Van der Waals (vdW) materials, composed of atomically thin layers bound by weak interlayer forces^1,2^, rose to prominence after the groundbreaking isolation of graphene, which was a transformative milestone in materials science. Over the past two decades, this diverse material family has served as a fertile platform for both fundamental discoveries and technological innovation^1–3^, owing to its distinctive optical^4^, electrical^5^, thermal^6^ and mechanical^7^ properties. In photonics, vdW materials have shown great promise due to their unique optical and electronic properties, such as strong optical nonlinearities^8,9^, pronounced excitonic resonances^10^ and tunable refractive indices^11^. These features have facilitated breakthroughs in light emission^12,13^, frequency conversion^14^, optical modulation^11^, polaritonic phenomena^15^, photodetection^16^ and spectroscopy^17,18^. In parallel, it is a continuing priority in integrated photonics to consolidate diverse optical functionalities (for example, nonlinear optical processing, amplification and modulation) onto a single chip as part of the progress towards the development of photonic integrated circuits^19^. In this context, vdW materials provide a compelling materials library for on-chip photonics^20,21^, as illustrated in Fig. 1a. Their atomically flat surfaces minimize optical scattering losses, and their unmatched compatibility with heterogeneous integration enables the construction of complex, vertically stacked architectures without lattice-matching constraints. Moreover, their extensive material library offers unprecedented flexibility for tailoring photonic and optoelectronic functions, further amplifying their appeal for highly integrated multifunctional systems.

Despite their considerable promise, existing approaches have largely confined vdW materials to auxiliary roles—typically as coated or embedded functional layers on top of conventional integrated photonic platforms—rather than employing them as structural building blocks. For example, graphene has been embedded as gate-tunable dispersive elements^22^ and nonlinear optical coatings^23^ in integrated photonics. Transition-metal dichalcogenide layers have been transferred onto silicon-based integrated devices to enable high-performance photodetection^24^ and modulation^25^. Although these studies highlight the substantial potential of vdW materials in nanophotonics and optoelectronics, their role remains largely limited and their full capacity for enabling next-generation photonic systems has yet to be realized.

Here we establish vdW materials as a viable material platform for low-loss nonlinear photonics. We introduce a generally applicable nanofabrication strategy based on focused ion beam (FIB) lithography with aluminium (Al) passivation, which enables high-resolution patterning across a broad range of vdW materials and their heterostructures. This method facilitates the fabrication of essential photonic components—including photonic crystals, metasurfaces, and microdisks—with nanometre-scale precision and well-preserved material quality to support high-quality integrated photonics. Of particular note, the fabricated microcavities support whispering gallery modes (WGMs) with quality (*Q*) factors exceeding 10^6^, spanning from the telecom to the visible regimes. These high-*Q* cavity resonance modes, combined with the strong intrinsic optical nonlinearity of vdW materials, enable highly efficient nonlinear optical processes, even under continuous-wave (CW) excitation, including second-harmonic generation (SHG), sum-frequency generation (SFG) and optical parametric amplification (OPA). Notably, the normalized SHG conversion efficiency is enhanced by four orders of magnitude compared with previously reported vdW-materials systems, and the generated signal is tunable over a full free spectral range (FSR). Our results establish a design paradigm for on-chip nonlinear photonics and position vdW materials not as supplementary layers but as potential foundational platforms for high-performance photonic circuits, with promising applications in reconfigurable photonics, nonlinear signal processing and quantum technologies.

## Fabrication of vdW microstructures and nanostructures for microcavity photonics

Diverse vdW materials provide a versatile platform for photonic and optoelectronic applications, with the promise of delivering tailored solutions beyond the limits of conventional monolithic photonics. With bandgaps spanning 0–6 eV, the vdW-materials family encompasses metals (for example, MXenes), semiconductors (for example, MoS_2_ and WS_2_) and insulators (for example, hexagonal boron nitride (h-BN)), thus enabling tailored heterostructures for diverse functionalities. Semiconducting transition-metal dichalcogenides exhibit high refractive indices advantageous for miniaturized photonic devices^26–33^. Many vdW materials also demonstrate superior nonlinear optical properties compared with traditional platforms like lithium niobate, with exceptionally high second- and third-order nonlinear optical susceptibilities (for example, GaSe and NbOCl_2_). These attributes potentially facilitate heterogeneous integration and empower new device designs beyond the reach of conventional photonic integrated circuits.

Notwithstanding these advantages, fabricating vdW materials into nanostructures suitable for photonic integration remains technically challenging. Standard top-down approaches—including electron-beam lithography followed by reactive-ion etching^34^ or direct femtosecond laser ablation^35^—often suffer from poor material compatibility. For reactive-ion etching, etching selectivity and surface quality are the main limitations due to the chemical inertness of certain vdW compounds. For example, fluorine-based gases, such as CF_4_, CHF_3_ and SF_6_, are effective in etching Mo- and W-based dichalcogenides (for example, refs. ^27,28,34,36–38^), but they are almost non-reactive with some XSe (where X = In, Ga and so on). This lack of reactivity leads to limited etch selectivity against photoresists and hard masks, resulting in poorly defined sidewalls and substantially hampering the ability to create clean, high-resolution optical structures. Femtosecond laser ablation, although material-agnostic in principle, introduces thermal damage and surface roughening, which degrades both structural and optical quality. These limitations hinder the scalability, fidelity and performance of vdW-based photonic components, especially in applications that rely on strong optical confinement or high-*Q* resonances.

**Extended Data Fig. 1:**
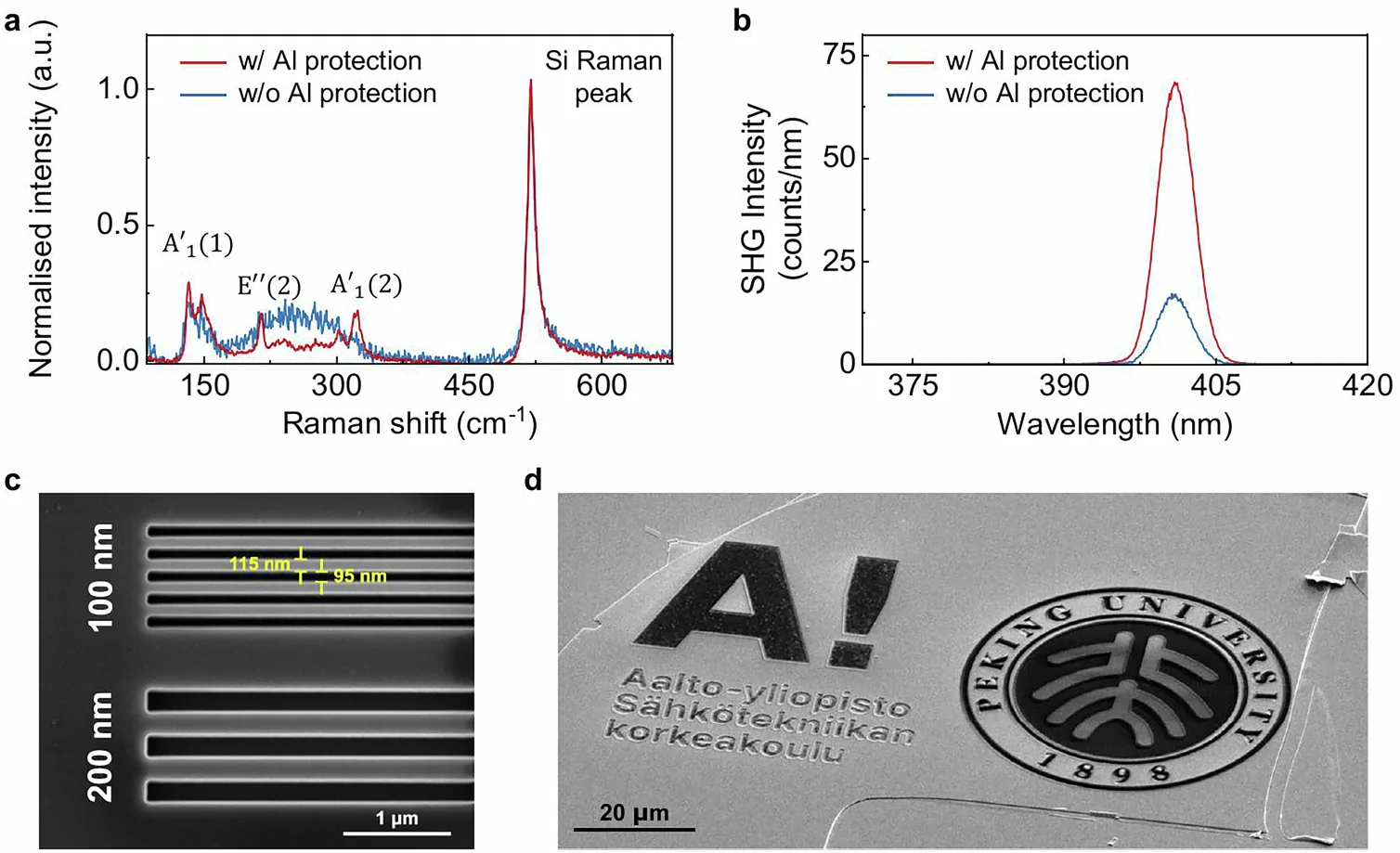
FIB patterning and Al protection method. **a**, Raman spectra measured from GaSe fabricated with and without Al passivation. **b**, Measured SH intensity from GaSe fabricated with and without Al passivation. The results underscore the critical role of Al passivation in preserving the vdW material properties during FIB lithography. **c**, Resolution characterisation of the fabrication technique. The resolution refers to the smallest achievable half-pitch of a line-space pattern with 50% duty cycle. The smallest achievable individual feature can be much smaller than the resolution, and is limited by the magnitude of process errors. **d**, Logos of Aalto University (left) and Peking University (right) patterned on vdW materials, showcasing the ability to define arbitrary shapes. The Raman and SHG results are obtained from free-space measurements (the measurement setup shown in Fig. S1), without involvement of structure resonances (for example, WGMs); the observed signal differences therefore originate solely from material degradation and serve to demonstrate the protective role of the Al film.

To overcome these challenges, we developed a generally applicable nanofabrication strategy based on FIB lithography with Al passivation (inset of Fig. 1a; see Methods for details). The fabrication process begins with the mechanical exfoliation of vdW flakes^3^, followed by a polydimethylsiloxane (PDMS)-assisted dry transfer^39^. A thin Al layer (~50 nm) is subsequently deposited, then direct-write FIB milling is applied to define arbitrary nanoscale patterns. The Al layer is finally removed by wet etching to reveal intact and well-patterned vdW structures (Supplementary Fig. 10). The Al layer serves two critical functions: protecting the underlying vdW materials from ion implantation damage^40,41^ and mitigating surface charging to prevent pattern deformation during FIB milling. The surface passivation is especially crucial for preserving the optical performance, as even a single Ga-ion imaging scan can introduce severe crystal defects and increase scattering losses in waveguide and resonator devices. We implemented free-space measurements to demonstrate the Ga-ion-induced material degradation (Extended Data Fig. 1a,b; the measurement set-up is shown in Supplementary Fig. 1). Raman spectroscopy and the second-harmonic (SH) intensity confirm that samples processed with Al passivation retain superior crystalline and optical properties compared with their unprotected counterparts. Under the with-Al protection condition, Raman peaks are better resolved and the SH intensity is four times higher than in the without-Al case, both reflecting the change of the intrinsic properties from the Ga-ion irradiation. We emphasize that we did not use fabricated structures (for example, WGM microcavities) in these free-space experiments, so that the observed signal differences arise solely from material degradation and serve to demonstrate the protective function of the Al film.

By primarily using the physical fabrication methods, we avoid using many hazardous chemical etchants that risk substrate damage, rendering this method applicable across a broad range of vdW materials. Our method provides accurate pattern alignment and can be used to fabricate vdW nanostructures spanning from single-material systems to diverse vertically stacked, free-selection vdW heterostructures. The resolution achieved (sub-100 nm, as demonstrated by the fabrication of the line-space patterns shown in Extended Data Fig. 1c and the linewidth histogram in Supplementary Fig. 11) is comparable with that from electron-beam lithography, while eliminating the need for a hard mask and greatly simplifying the process flow.

Using the described fabrication protocol, we created a series of vdW-based photonic nanostructures—microdisks, photonic crystals and arrays of triangular resonators—from various materials, such as h-BN, MoS_2_, GaSe and NbOCl_2_ (Fig. 1b). The fabrication of complex geometries, including stacked heterostructures (for example, the MoS_2_/VOCl_2_-stacked microdisk in Fig. 1b) and institutional logos (see our universities’ logos in Extended Data Fig. 1d), further highlights the precision and generality of the method. Taken together, our FIB-based fabrication strategy provides a robust, material-independent route for high-quality vdW photonic structures. It outlines a possible route towards reconfigurable and multifunctional vdW photonic integrated circuits that leverage the full material versatility of this platform.

## High-*Q* vdW microcavities

**Fig. 2:**
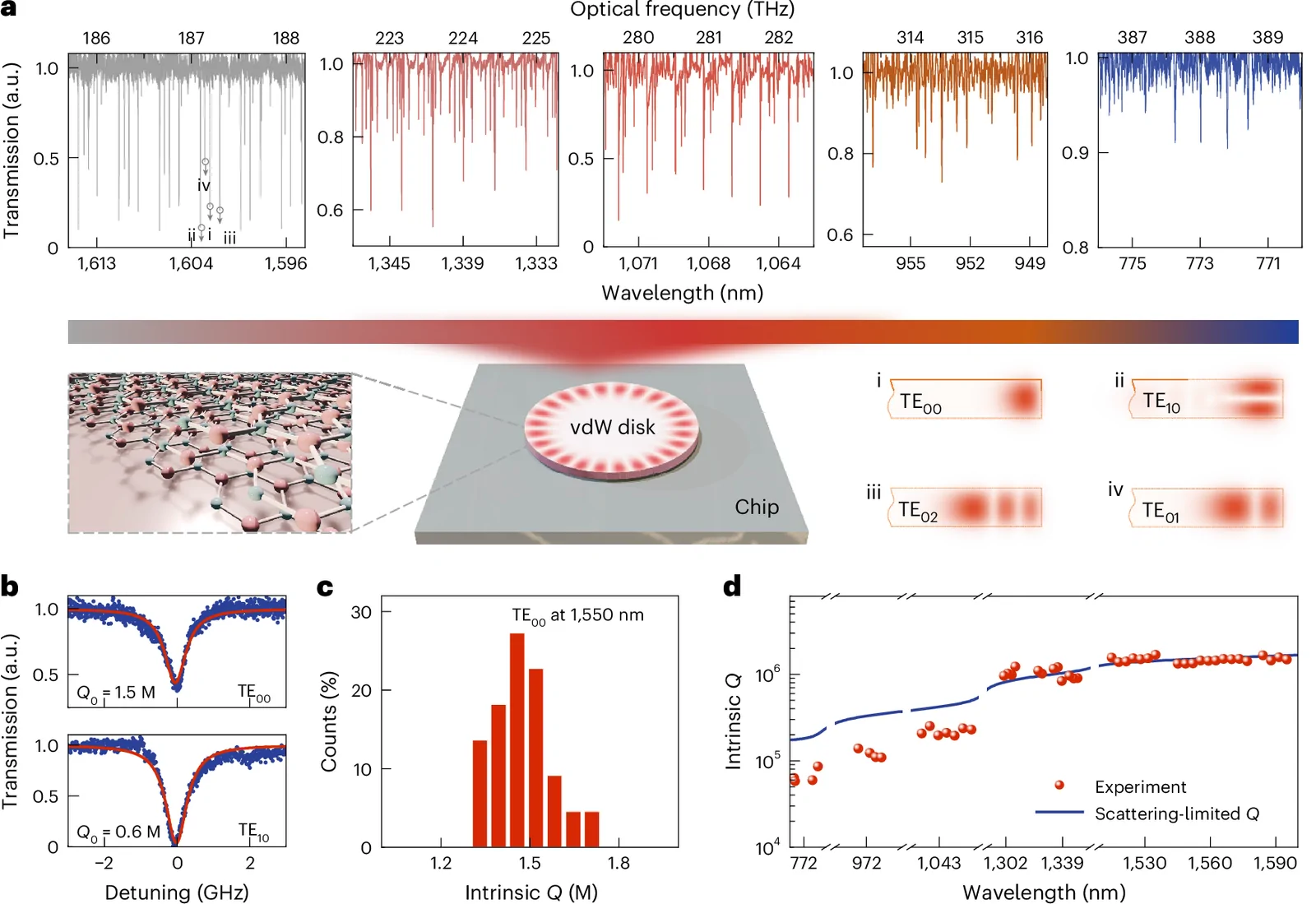
vdW whispering gallery microcavities with an ultrahigh *Q* factor. **a**, Top: broadband transmission spectra measured in different wavelength bands, showing cavity resonances across a wide spectral range. Grey arrows in the first spectrum mark four different mode families (TE_00_, TE_10_, TE_02_ and TE_01_). Bottom left: schematic of the structure of the vdW material. Bottom middle: schematic of a vdW microcavity on a single chip supporting WGMs. Bottom right: numerically simulated electric field distributions corresponding to the WGM families (i to iv) shown in the first spectrum in the top panel. **b**, Typical transmission spectra of TE_00_ and TE_10_ modes for *Q*-factor extraction. *Q*_0_, the intrinsic *Q* factor. **c**, Statistical distribution of the intrinsic *Q* factor of TE_00_ modes in the telecom band. **d**, Wavelength-dependent intrinsic *Q* factors measured over several spectral bands compared with theoretical predictions based on scattering loss from surface roughness. Source data

The ability to fabricate ultralow-loss optical microcavities is essential for advancing integrated photonics. Among various resonant structures, WGM microcavities stand out for their ability to confine light within small mode volumes while maintaining ultrahigh *Q* factors, making them an excellent platform for enhancing light–matter interactions. To evaluate the quality of our fabrication approach, we constructed microcavities using various vdW materials transferred onto quartz substrates. The microdisks were designed with thicknesses of hundreds of nanometres and with radii spanning tens of micrometres to optimally support WGMs via total internal reflection. The *Q* factor, defined as the ratio of the energy stored in the cavity to the energy lost per optical cycle, was measured by coupling light into the microdisk via a tapered optical fibre with a U-shaped configuration (Supplementary Fig. 2). This set-up enables evanescent excitation and the comprehensive characterization of the WGMs. We used wavelength-tunable CW lasers to scan across broad spectral ranges, from the visible to the telecom bands. Transmission spectra for h-BN microdisks across these wavelengths are shown in Fig. 2a. At telecom bands, four transverse electric (TE) mode families are clearly identified, TE_00_, TE_10_, TE_02_ and TE_01_. The corresponding field distributions simulated using COMSOL Multiphysics are plotted in the bottom right corner of Fig. 2a. Only TE modes were observed experimentally due to the high in-plane refractive index of h-BN (*n*_∥_ ≈ 2.20), which strongly confines light in the cavity plane. By contrast, transverse magnetic modes, which have their electric field mainly oriented perpendicular to the microcavity plane, have a lower refractive index (*n*_⊥_ ≈ 1.58) that fails to provide sufficient optical confinement, resulting in leaky modes with substantially reduced *Q* factors.

A closer view of the transmission spectra around the two modes TE_00_ and TE_10_ in the 1,550-nm band is shown in Fig. 2b. From these spectra, fairly high intrinsic *Q* factors of around 10^6^ were obtained (see Methods and Supplementary Section IV for details of the characterization technique). Furthermore, a statistical survey of TE_00_ modes across the telecom band confirmed that these *Q* values were consistently achieved (Fig. 2c). Such high *Q* values, corresponding to a propagation loss of approximately 0.35 dB cm^−1^, highlight the benefits of the atomic-level surface and smooth sidewalls of the microdisks. At this level, the scattering loss becomes the dominant contributor to the total optical loss, outweighing both material absorption and radiation leakage^42^. We experimentally observed a wavelength-dependent trend in *Q*, with higher *Q* values at longer wavelengths (Fig. 2d), consistent with theoretical predictions based on surface roughness scattering (Supplementary Section V). This trend underscores the critical role of our fabrication precision in reducing the scattering loss and enabling high-*Q* microcavities, while also highlighting the possibility of further improvements towards even better devices.

**Extended Data Fig. 2:**
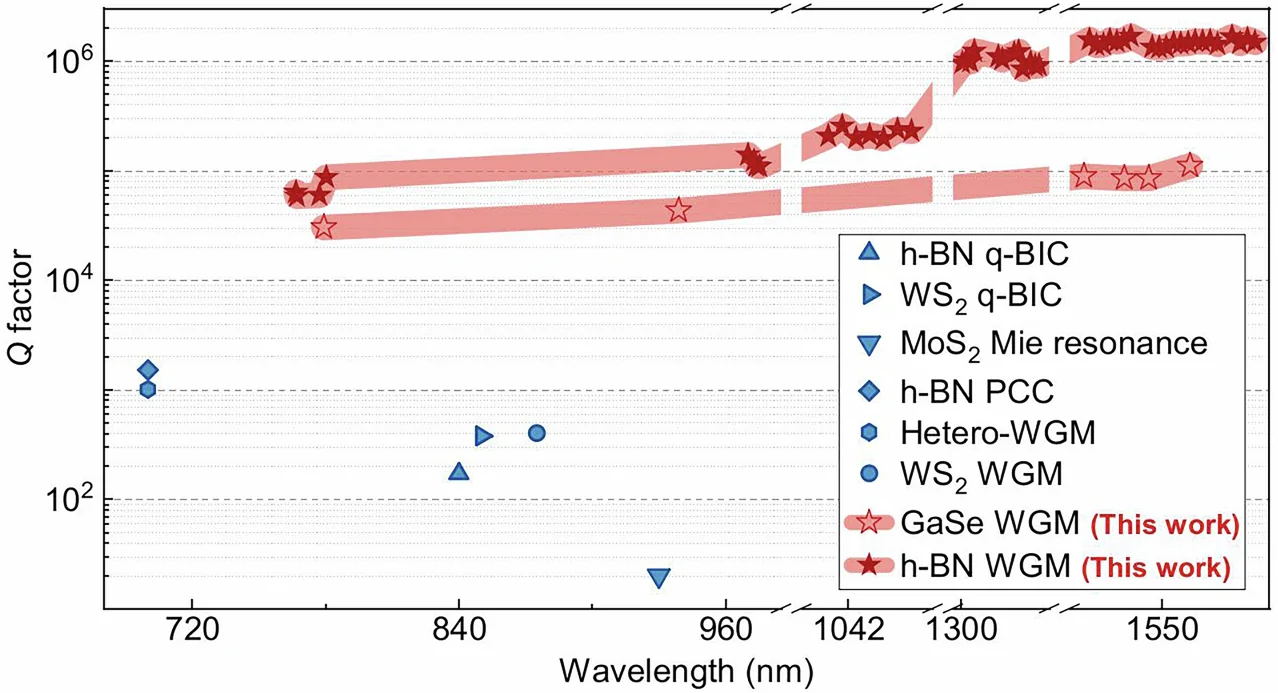
Comparison of realised Q factors of resonators on vdW material platforms. Comparison encompasses multiple resonant modes: quasi-bound states in the continuum (q-BIC)^26,56^, photonic crystal cavity (PCC)^57^, Mie resonator^28^ and WGM cavity^34,58^, evaluated across the spectral range from 680 - 1600 nm.

The demonstration of vdW microdisk resonators with *Q* > 10^6^ confirms that our fabrication method enables vdW materials to fully exploit their intrinsic optical property advantages. The demonstrated performance surpasses that of many previous vdW-materials resonant systems by three orders of magnitude (Extended Data Fig. 2 and Supplementary Table 1). This high level is sustained across densely spaced modes spanning telecom to near-visible wavelengths. These microcavities provide a foundational building block for diverse photonic functionalities, including quantum light generation and low-threshold lasing. Note that h-BN has a bandgap of ~6 eV, thus offering a wide operational transparency window; however, the characterization of the h-BN microdisks beyond the telecom to near-visible ranges is limited by the wavelength coverage available in our laboratories. Nevertheless, the ability of various vdW materials to operate across a broad wavelength range—from infrared to ultraviolet—enhances their versatility for integration into multiband photonic systems.

## Highly efficient CW-driven tunable SHG

**Fig. 3:**
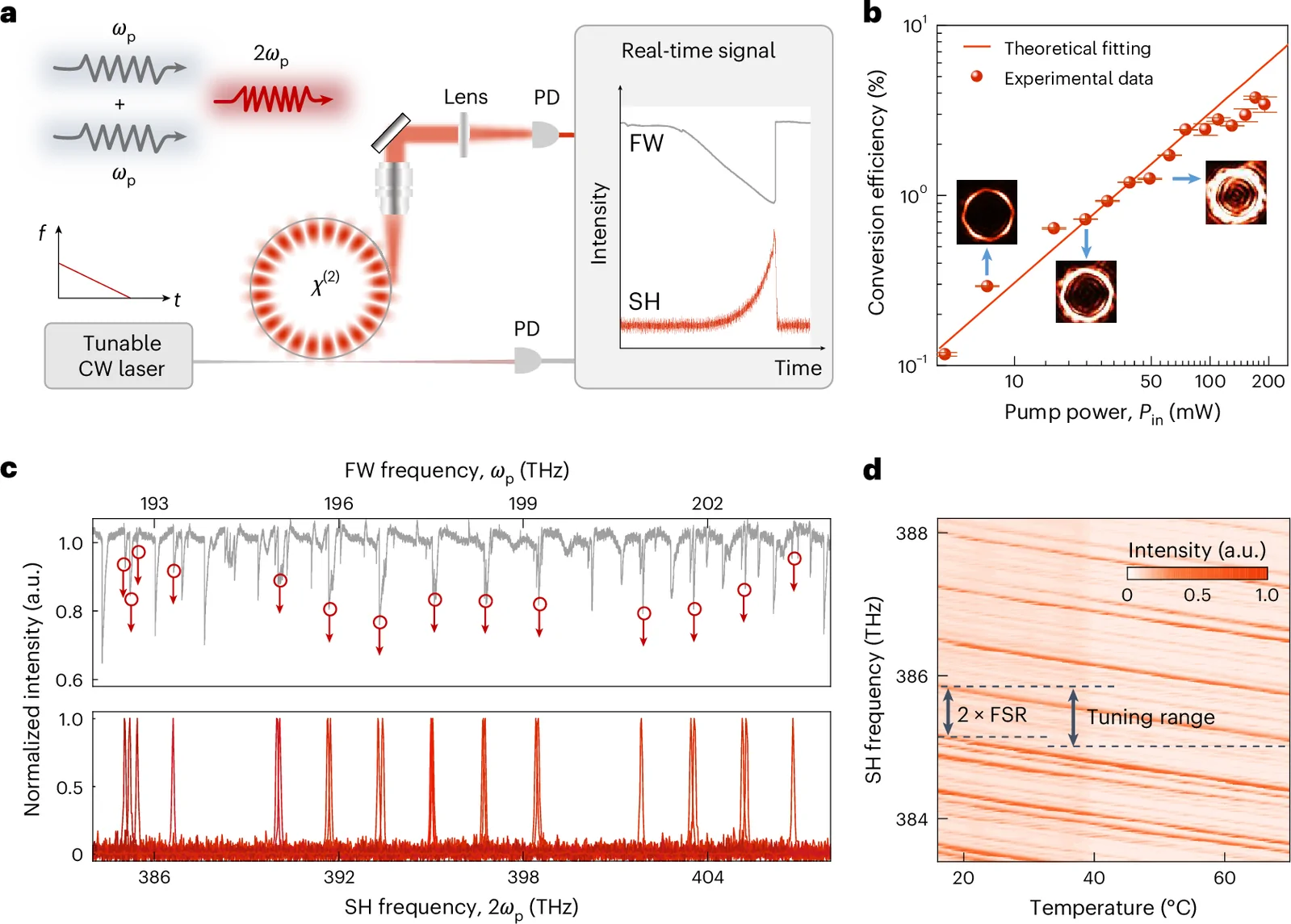
Highly efficient CW-driven tunable SHG in a GaSe microcavity. **a**, Experimental set-up for SHG measurement. A tunable CW laser is coupled via a fibre taper. Inset: SHG process and typical time-scan results. *f*, frequency. **b**, SHG conversion efficiency as a function of pump power. The results are the mean of three independent measurements. Insets: top-view images of the microdisk showing SH emission at different pump power levels. **c**, Broadband transmission FW spectrum (top) and the corresponding SH signal (bottom). Red circles mark the FW frequencies that generate SH signals. **d**, Thermally tunable SH frequency as a function of temperature ranging from 16 °C to 70 °C. Twice the FSR in the FW band (~0.70 THz) and the thermal-tuning SH range (~0.76 THz) are indicated for comparison. The GaSe microcavity radius was ~15 μm in **b**, 20 μm in **c** and 60 μm in **d**. PD, photodetector. Source data

Nonlinear optical processes lie at the heart of integrated photonics, as they enable frequency conversion, quantum light generation and reconfigurable signal processing. Leveraging the high-*Q* feature mentioned above, accompanied by the exceptionally high nonlinearity of vdW materials, we demonstrated highly efficient *χ*^(2)^- and *χ*^(3)^-nonlinear optical processes. We first demonstrated SHG, one of the most essential nonlinear optical processes, using GaSe microcavities with high second-order optical nonlinearity^43^. To do this, a CW fundamental wave (FW) was injected into the GaSe microdisk through a U-shaped fibre taper, and its frequency *ω*_p_ was gradually downtuned across the cavity modes. Owing to the interplay between the Kerr and thermo-optic effects, the transmission spectra exhibited characteristic non-Lorentzian ‘thermal triangle’ features (Fig. 3a). As the pump wavelength approached resonance, the resonance-enhanced intracavity power intensity led to a steep rise in SH emission at 2*ω*_p_, which was collected vertically through an objective lens.

The SHG efficiency, measured as a function of pump power, exhibited a linear scaling in the weak-pump regime (Fig. 3b), consistent with theoretical expectations for the undepleted pump regime^44^. With increasing pump power (>100 mW), the efficiency slightly deviated from the theoretical line, which is attributed to a thermally induced change in the fibre–cavity coupling condition as the cavity temperature rises. Real-space imaging of the microdisk confirmed the spatial localization of the SH signal at the cavity perimeter (insets of Fig. 3b). Notably, the highest absolute SHG efficiency reached ~3.7 ± 0.1% at ~172 mW. The normalized efficiency in the undepleted regime was ~30% W^−1^, an improvement of four orders of magnitude over previously reported vdW systems^45,46^. This notable improvement is attributed to the high *Q* of the FW cavity mode, as SHG efficiency scales quadratically with the *Q* factor of the FW mode^44^.

Further, we demonstrated broadband SHG behaviour. By downscanning the FW frequency ranging from ~204 THz to 192 THz (correspondingly from 1,470 nm to 1,560 nm), we obtained an SH signal ranging from ~408 THz to 384 THz, as plotted in the top and bottom panels of Fig. 3c, respectively. The resulting SH peaks appeared at typical intervals of about 1.75 THz, corresponding to twice the FSR of the TE_00_ cavity mode family at the telecom band. Also note that some frequencies corresponding to FW resonances, for example, those near 390 THz and 400 THz, did not produce observable SHG peaks. This phenomenon is attributed to two primary factors: (1) mode crossings between distinct mode families led to reduced coupling efficiency and (2) momentum mismatch between the pump and SH light generated within the microcavity impeded efficient SHG.

**Extended Data Fig. 3:**
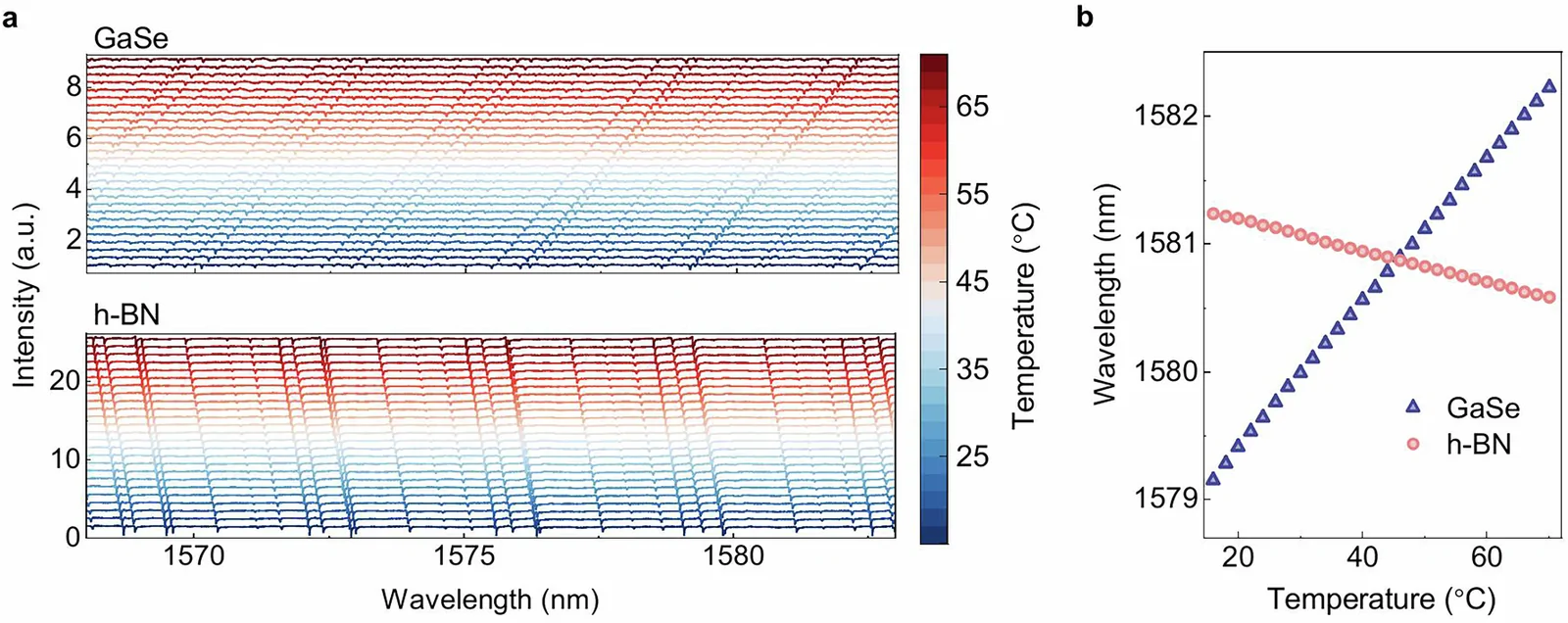
Thermal tunability of WGMs with different vdW materials. **a**, Broadband transmission spectra for GaSe and h-BN microdisks at varying substrate temperatures. **b**, Temperature dependence of the resonance wavelength for the GaSe and h-BN microdisks extracted from **a**.

The high-efficiency process was achieved without relying on double resonance or strict phase matching but, instead, through the combination of ultrahigh *Q* factors, strong second-order nonlinearity and large effective interaction volumes in vdW microcavities. These results indicate strong potential for further improvement once the phase-matching and double-resonance conditions are fully engineered. Moreover, this process is good for realizing a high-efficiency broadband nonlinear process owing to the released mode-sensitive phase-matching condition. Indeed, using the same GaSe material system with a large thermo-optic coefficient, we achieved thermally tunable SHG in a microdisk resonator, which presented obvious changes in the effective refractive index with temperature (Extended Data Fig. 3). This thermal tuning shifted the resonant frequencies of the cavity modes, thereby enabling tunability of broadband SHG. Figure 3d presents the collected results of generated SH light. The SH frequency spanned from ~383 THz to 388 THz for different substrate temperatures (16 °C to 70 °C). Notably, temperature tuning enabled a continuous SHG frequency scan of ~0.76 THz, which exceeds twice the FSR in the FW band (~0.70 THz). This indicates the potential of the GaSe microdisk to generate a widely tunable SHG signal within its optical transparency window. Such temperature-based control offers substantial potential for on-chip photonic applications, including wavelength-agile light sources, reconfigurable photonic circuits and integrated spectroscopy.

## CW-driven SFG and OPA

Nonlinear processes, such as SFG and OPA, are critical for wavelength conversion, coherent signal generation and quantum optics. These processes typically require precise phase matching and high pump powers, which are difficult to achieve under CW excitation in vdW-materials platforms. Here we demonstrate both CW-driven SFG and OPA in vdW microcavities, which are enabled by high-*Q* confinement and strong intrinsic optical nonlinearity.

**Fig. 4:**
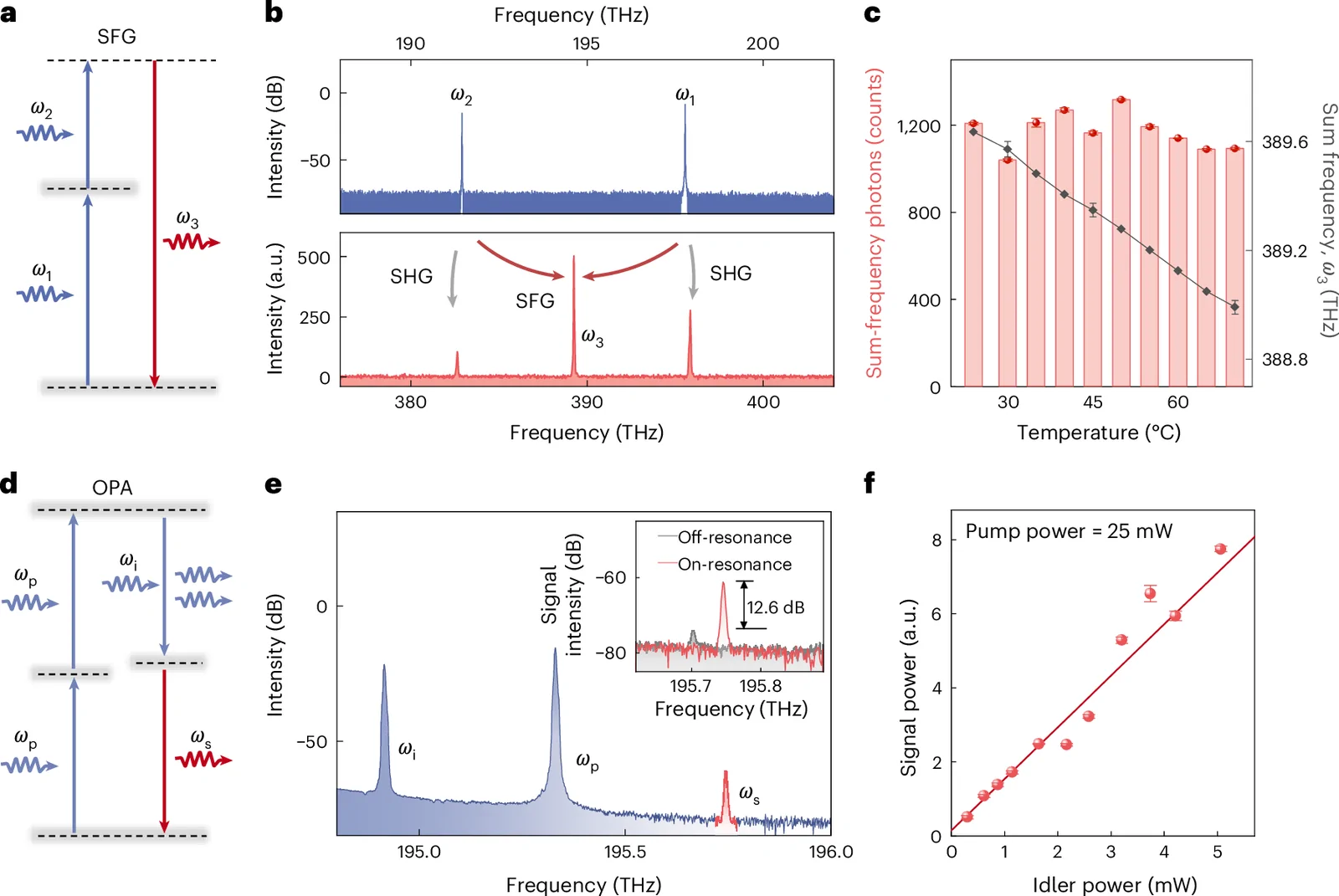
Demonstration of CW-driven SFG and OPA processes. **a**, Energy-level diagram illustrating an SFG process in a GaSe microcavity. Two input fields at *ω*_1_ and *ω*_2_ (blue) generate a sum-frequency signal at *ω*_3_ = *ω*_1_ + *ω*_2_ (red). **b**, Spectra showing the input FW beams (top) and the generated SHG and SFG signals (bottom). **c**, Thermally tunable SFG signal intensity (red bars) and its corresponding SFG frequency tuning (grey curve) over a temperature range of 20–73 ^∘^C. The radius of the GaSe microcavity was ~20 μm. Data are shown as mean ± standard deviation from four independent measurements. **d**, Energy-level diagram of a *χ*^(3)^ OPA process in an h-BN microcavity. *ω*_p_, *ω*_i_ and *ω*_s_ denote the frequencies of the pump, idler and signal, respectively. **e**, Spectrum showing the pump, idler and signal light. Inset: zoom-in of the generated signal power when the pump light is on-resonance (red) and off-resonance (grey) with respect to the cavity mode. **f**, Dependence of the output signal power on the input idler power, with the pump power fixed at 25 mW. The radius of the h-BN microcavity was ~50 μm. Data are shown as mean ± standard deviation from three independent measurements. Source data

SFG is a second-order nonlinear optical process wherein two input photons of frequencies *ω*_1_ and *ω*_2_ interact to produce a photon at the sum frequency (*ω*_3_ = *ω*_1_ + *ω*_2_), as schematically illustrated in Fig. 4a. In our experiment, two independent CW lasers were tuned to resonate with the distinct modes of a GaSe microdisk at approximately 191.5 THz and 197.8 THz, respectively. These pumps were coupled into the microdisk via a tapered fibre and excited separate TE-mode resonances. The resulting SFG signal appeared at ~389.3 THz, and the accompanying SHG signals were observed near 382.7 THz and 395.9 THz (Fig. 4b). The observed slight deviations between the expected and measured frequencies are attributed to two factors: (1) thermal and Kerr-induced resonance shifts arising under a high intracavity intensity and (2) imperfect fulfilment of the frequency-matching condition. The clear spectral features confirm the successful generation of both SFG and SHG within the same microcavity. Notably, the SFG output remained robust under thermal tuning from 20 °C to 73 °C as it maintained a consistent signal intensity of ~1,100 counts over a tuning range of ~0.7 THz (Fig. 4c). This robust signal benefitted from two pump beams independently resonating with separate cavity modes, and the SFG photon was not confined by a narrow microcavity resonance.

Further, we exploited the *χ*^(3)^ nonlinearity of h-BN in a separate microdisk resonator to demonstrate the OPA process. In that process, two pump photons at frequency *ω*_p_ and one idler photon at *ω*_i_ generated a signal photon at *ω*_s_ = 2*ω*_p_ − *ω*_i_ while simultaneously amplifying the idler wave (Fig. 4d). Experimentally, a strong pump at ~195.3 THz and a weaker idler at ~194.9 THz were coupled into the cavity. When the pump wave was on-resonance with a cavity mode, a new signal emerged near 195.7 THz (the red peak in Fig. 4e). A comparison between the on- and off-resonance conditions revealed a signal enhancement of ~12.6 dB (inset of Fig. 4e), confirming the resonance-assisted parametric gain. The output signal power exhibited a linear dependence on the idler input power, as expected for the OPA process (Fig. 4f). This demonstration establishes vdW microdisks as a viable platform for CW-driven nonlinear optical amplification. However, this amplification process is, at present, limited by a large detuning, which originates from the non-ideal cavity dispersion design. Implementing dispersion engineering to achieve the desired dispersion condition while taking the Kerr modulation into account could be a pathway to overcoming this limitation and enhancing the process.

## Conclusion

In conclusion, we have introduced a high-precision nanostructuring technique for a wide range of vdW materials that enables the fabrication of diverse photonic components with exceptional optical quality. As a representative example, we realized h-BN microcavities supporting WGMs with intrinsic *Q* factors exceeding 10^6^. Exploiting the high-*Q* resonances in a GaSe microcavity, we demonstrated CW-driven SHG with full FSR tunability. We finally reported efficient SFG and OPA, all achieved with CW excitations, an important advance over previous ultrafast-pulsed implementations. These findings address key limitations in nonlinear optical gain for vdW-based photonics and open avenues for energy-efficient light conversion on-chip.

Looking ahead, further optimization of the fabrication process to achieve higher *Q* cavities (Supplementary Section XIII), combined with carefully engineered cavity mode dispersion through geometric design and effective refractive index modulation, could substantially boost nonlinear optical performance under phase-matching conditions^44,47,48^. By combining high-*Q* resonances, exceptional nonlinearity and the potential for periodic poling^49^, vdW-based resonant platforms hold substantial promise for applications such as quantum light sources^50^, quantum logic gates^51^ and ultra-sensitive sensors^52^. Beyond these, the integration of quantum emitters with vdW cavities offers a pathway to cavity-quantum electrodynamic regimes that would enable spontaneous emission control and strong-coupling dynamics demonstrations^29,53,54^. Furthermore, coupling ultralow-loss vdW structures with other resonant modes, such as plasmonic, phononic and excitonic platforms, may unlock new physical phenomena and application frontiers^55^. Overall, our results establish vdW materials not merely as functional overlays but as foundational building blocks for next-generation integrated photonics, thus opening pathways for exploring light–matter interactions and for realizing scalable, high-performance, versatile photonic devices with excellent capabilities.

## Methods

### Device fabrication

The VdW materials used in this study were sourced from 2D Semiconductors and mechanically exfoliated onto PDMS substrates. Selected flakes were transferred onto quartz substrates using a deterministic dry-transfer method^3^. Atomic force microscopy (Bruker Dimension Icon) was used to characterize flake thickness. Before nanofabrication, samples were annealed under ~10^−5^ mbar at 170 °C for 2 h to improve surface cleanliness and adhesion. Then the samples were passivated with a 50-nm Al layer using physical vapour deposition (MASA IM-9912). The vdW materials were patterned via FIB (Helios Nanolab 600) using a programmed milling function (gallium ion beam). Finally, the Al passivation layer was removed by wet etching in dilute AZ 351B (Merck).

### *Q*-factor characterization

The *Q* factors of the disk microcavities were measured across a broad spectral range. Tunable CW lasers were employed in six spectral regions (770 nm, 980 nm, 1,064 nm, 1,330 nm, 1,460 nm and 1,550 nm; DLC CTL series, TOPTICA Photonics; TLB-6712-P, Newport). Calibration was performed using a fibre-based Mach–Zehnder interferometer. The laser was frequency-modulated and injected into the microdisk through a tapered fibre. A Lorentzian-type transmission spectrum was observed near the resonance frequency. The corresponding linewidth *κ* was read over the scanning time with the calibrated Mach–Zehnder interferometer, which enabled us to extract the loaded *Q* factor *Q* = *ω*/*κ*, where *ω* is the resonance frequency. The intrinsic *Q* factors were extracted via the loaded linewidth and the coupling efficiency, the detailed operation of which is given in Supplementary Section IV along with the calibration method.

### Nonlinear optical processes in a microcavity

Three nonlinear optical processes—SHG, SFG and OPA—were demonstrated in this work. SHG operates in a GaSe microdisk with second-order nonlinearity under the non-depletion regime, with the process further enhanced by cavity mode confinement. The coupled-mode equation for the pump mode can be described as

1 $$\frac{{\rm{d}}a}{{\rm{d}}t}=\mathrm{i}{\varDelta }_{{\rm{p}}}a-\frac{{\kappa }_{{\rm{p}}}}{2}a-{\rm{i}}\sqrt{{\kappa }_{\mathrm{p,ex}}}{a}_{\mathrm{in}},$$

where *Δ*_p_ denotes the pump-cavity detuning and *κ*_p_(*κ*_p,ex_) represents the total (external coupling) loss of the pump mode. The SH signal power can be written as

2 $$| b{| }^{2}=M\frac{2{{\rm{\pi }}}^{2}{\omega }_{{\rm{s}}}^{2}{\left|{\chi }^{(2)}\right|}^{2}{Q}_{{\rm{p}}}^{2}}{{\epsilon }_{0}{c}^{3}{n}_{{\rm{p}}}^{2}{n}_{{\rm{s}}}{V}_{\mathrm{eff}}^{2}}| a{| }^{4},$$

where *M* evaluates the field overlap between the FW mode and the SH free-space propagating field, ${a}_{{\rm{in}}}=\sqrt{{\kappa }_{{\rm{p,ex}}}{P}_{{\rm{in}}}/{\omega }_{{\rm{p}}}\hslash }$ represents the injected photon number. *ϵ*_0_ is the vacuum permittivity and ℏ is the reduced Plank constant. *Q*_p_ and *V*_eff_ denote the quality factor of the pump mode and the effective mode volumn. *n*_p_ (*n*_s_) represents the effective refractive index of the pump (SH) light. SFG is realized using dual CW pumps tuned to distinct cavity modes, with both pump fields governed by equation (1) under the non-depletion regime. As for the OPA process with the third-order nonlinearity within an h-BN microdisk, the coupled-mode equations are

3 $$\begin{array}{l}\frac{{\rm{d}}a}{{\rm{d}}t}={\rm{i}}{\varDelta }_{{\rm{p}}}a-\frac{{\kappa }_{{\rm{p}}}}{2}a-2{\rm{i}}g{a}^{* }bc-{\rm{i}}\sqrt{{\kappa }_{\mathrm{p,ex}}}{a}_{\mathrm{in}},\\ \frac{{\rm{d}}b}{{\rm{d}}t}={\rm{i}}{\varDelta }_{{\rm{i}}}b-\frac{{\kappa }_{{\rm{i}}}}{2}b-{\rm{i}}g{a}^{2}{c}^{* }-{\rm{i}}\sqrt{{\kappa }_{\mathrm{i,ex}}}{b}_{\mathrm{in}},\\ \frac{{\rm{d}}c}{{\rm{d}}t}={\rm{i}}{\varDelta }_{{\rm{s}}}c-\frac{{\kappa }_{{\rm{s}}}}{2}c-{\rm{i}}g{a}^{2}{b}^{* },\end{array}$$

where *a* (a*), *b* (b*) and *c* (c*) denote the photon number amplitude (and their conjugates) for the pump, idler and signal modes, respectively, and ${b}_{{\rm{in}}}=\sqrt{{\kappa }_{{\rm{i,ex}}}{P}_{{\rm{in}}}/{\omega }_{{\rm{i}}}\hslash }$ represents the injected idler photon number. *g* is the single-photon coupling strength. *Δ*_i_ (*Δ*_s_) denotes the idler (signal)-cavity detuning, *κ*_i_ (*κ*_s_) represents the total loss of the idler (signal) mode, and *κ*_i,ex_ is the external coupling loss for the idler mode.

### Reporting summary

Further information on research design is available in the Nature Portfolio Reporting Summary linked to this article.

## Online content

Any methods, additional references, Nature Portfolio reporting summaries, source data, extended data, supplementary information, acknowledgements, peer review information; details of author contributions and competing interests; and statements of data and code availability are available at 10.1038/s41563-026-02574-x.

## Supplementary information

Supplementary Information Supplementary Sections I–XIII, Figs. 1–11, and Tables 1 and 2.

Reporting Summary

## Source data

Source Data Fig. 2 All data are included.

Source Data Fig. 3 All data are included in two sheets.

Source Data Fig. 4 All data are included.

## Data Availability

Source data are provided with this paper.
