# Supplementary material for: All-van der Waals microcavities for low-loss nonlinear photonics
Source: Nat Mater. 2026 Apr 13;25(9):1549–56. doi: 10.1038/s41563-026-02574-x (PMC13518243; doi:10.1038/s41563-026-02574-x)
Supplement: Supplementary file 1 [file 41563_2026_2574_MOESM1_ESM.pdf]

nature materials

Supplementary information

https://doi.org/10.1038/s41563-026-02574-x

All-van der Waals microcavities for low-loss nonlinear photonics

In the format provided by the authors and unedited

CONTENTS

I. Comparison of fabrication techniques for van der Waals material structures 2

II. Aluminium passivation 4

III. Coupling technique of microcavities 5

IV. Calibration of $\boldsymbol{Q}$ factors via fibre-based Mach-Zehnder interferometer (MZI) 7

V. Theoretical model of scattering loss 10

VI. Comparison of the SHG efficiency among vdW systems 12

VII. SHG efficiency calibration method 14

VIII. Damage threshold of GaSe and the limit of the SHG efficiency 15

IX. Thermal management for our high-Q, high-power devices 16

X. Noise figure (NF) analysis for the OPA process 17

XI. Sidewall roughness and surface effect induced by Al removal 18

XII. Standard deviation of FIB milling 20

XIII. Discussion on FIB-based vdW material patterning 21

References 24

I. COMPARISON OF FABRICATION TECHNIQUES FOR VAN DER WAALS MATERIAL STRUCTURES

Method & Mask	M.S. L.S.	Applied Materials	Structure	Size (µm)	Q factor	Application	Ref.	Year
EBIE FIB maskless	No -	h-BN	Photonic crystal cavity	-	∼2100	Single photon emitter	[1]	2018
RIE(SF_6) polymer EBIE Tungsten	Yes Yes	h-BN	PIC components	-	∼500 ∼1500	PIC components	[2]	2018
RIE (CHF_3+Ar) polymer	Yes Yes	WS_2	Mie resonator	0.22-0.4	-	Strong coupling	[3]	2019
FIB polymer	No -	WS_2	Mie resonator Nanodisk array	0.4-1	-	Enhance SHG by ∼ 50×	[4]	2020
RIE(SF_6) Aluminium	Yes Yes	h-BN	WG-coupled disk resonator	8.5	10^3	Couple excitons to WGM	[5]	2020
RIE(SF_6+ CHF_3+Ar) Aluminium	Yes Yes	MoS_2	Mie resonator Metasurface	0.2-0.24	-	Directional SHG & THG	[6]	2021
RIE (SF_6+Ar) polymer	Yes Yes	h-BN	Waveguide	25	-	Single photon emitters	[7]	2021
FLP -	No Yes	γ-InSe	Microdisk	20-30	-	WGM-based NIR lasing at RT	[8]	2021
RIE (CF_4+O_2) polymer	Yes Yes	WS_2	Microdisk	3-5.3	∼400	Lasing at RT	[9]	2022
RIE(Ar) polymer	No Yes	WS_2	Nanodisk	0.3-0.8	-	Exciton emitters at 4.2 K	[10]	2022
RIE(SF_6) polymer	Yes Yes	2H-MoS_2	Mie resonator	0.3 & 0.55	-	Enhance SHG by ∼ 23×	[11]	2022
RIE(CHF_3 & SF_6) polymer	Yes Yes	WS_2	Double-pillar nanoantennas	∼0.5-1	-	Purcell enhancement by ∼ 240×	[12]	2022
RIE(CF_4 polymer	Yes Yes	2H-MoS_2	Waveguide	10-50	-	PIC components	[13]	2022
RIE(BCl_3+ Cl_2)(C_2H_4+ SF_6) Aluminium	Yes Yes	2H-WS_2	Waveguide	0.25-0.45	-	PIC components Coupler	[14]	2023
RIE hard mask	Yes Yes	MoS_2	Waveguide Grating Coupler	20	-	PIC components	[15]	2023
RIE(CF_4 +O_2) polymer	Yes Yes	WS_2	Waveguide	0.6-0.8	-	Supporting polariton	[16]	2023

**Method & Mask**	**M.S. L.S.**	**Applied Materials**	**Structure**	**Size (µm)**	***Q***	**Application**	**Ref.**	**Year**
RIE(SF_6) metal polymer	Yes Yes	h-BN	Waveguide	∼50	2000-4000	PIC and quantum components	[17]	2023
RIE(SF_6) Gold	Yes Yes	h-BN	Metasurface	0.36	150-340	Photonic qBIC	[18]	2023
RIE (CHF_3+Ar) polymer	Yes Yes	WS_2, ReS_2, $TaSe_2$	PIC components Nanostructures	-	-	PIC components Nanophotonics	[19]	2023
RIE(CHF_3) polymer	Yes Yes	3R-MoS_2	Nanodisk	0.26-1	∼11-20	Enhance SHG by $10^2\times$ to $10^4\times$	[20]	2024
RIE(SF_6) Gold	Yes Yes	WS_2	Metasurface	35	20-370	Enhance light-matter interaction for hybridization of photonic and excitonic modes	[21]	2024
RIE(SF_6) Gold	Yes Yes	h-BN	Metasurface	0.36	120-180	Photonic qBIC	[22]	2024
FIB Chromium	No No	$NbOCl_2$	Mie resonator	0.5 & 1	-	Enhance SHG by $25\times$	[23]	2024
FIB Aluminium	No -	GaSe, MoS_2, $NbOCl_2$, h-BN	Microdisks PIC components	0.2-120	$> 10^6$	CW-based NLO: SHG by $10^4\times$ SFG & OPA	This work	2026

Table S1: **Different fabrication methods for vdW nanostructures and their corresponding device performance.** *Q* factor, quality factor; M.S., material sensitivity; L.S., Large scale; EBIE, electron beam induced etching; FIB, focused ion beam; RIE: reactive ion etching; PIC, photonic integrated circuit; SHG, second harmonic generation; WGM, whispering gallery mode; THG, third harmonic generation; FLP, femtosecond-laser printing; NIR, near infrared; RT, room temperature; qBIC, quasi-bound states in the continuum; CW, continuous-wave; NLO, nonlinear optics.

II. ALUMINIUM PASSIVATION

We demonstrate the benefits of aluminium (Al) passivation in our fabrication process by comparing the SHG and Raman spectra of passivated and non-passivated GaSe reference samples, as shown in Extended Data Figs. 1**a** and **b**.

For the Raman data, we attempt to fabricate 15-µm-diameter disks on both samples. As expected, the unprotected sample charges quite a bit, and therefore, the pattern drifts during exposure, and the disk is severely distorted. The sample with Al protection shows nice crystalline behaviour matching the expected Raman peaks of GaSe (A'$_1$(1) at 134 cm^{-1}, E"(2) at 214 cm^{-1}, and A'$_1$(2) at 308 cm^{-1}, values from literature[24]). By contrast, the unprotected sample exhibits a broad amorphous Raman response superimposed on the crystalline signal, which originates from deeper regions within the sample. We attribute this to the incident 30 kV Ga$^+$ ion beam, which is expected to amorphize the top 30–50 nm of the flake.

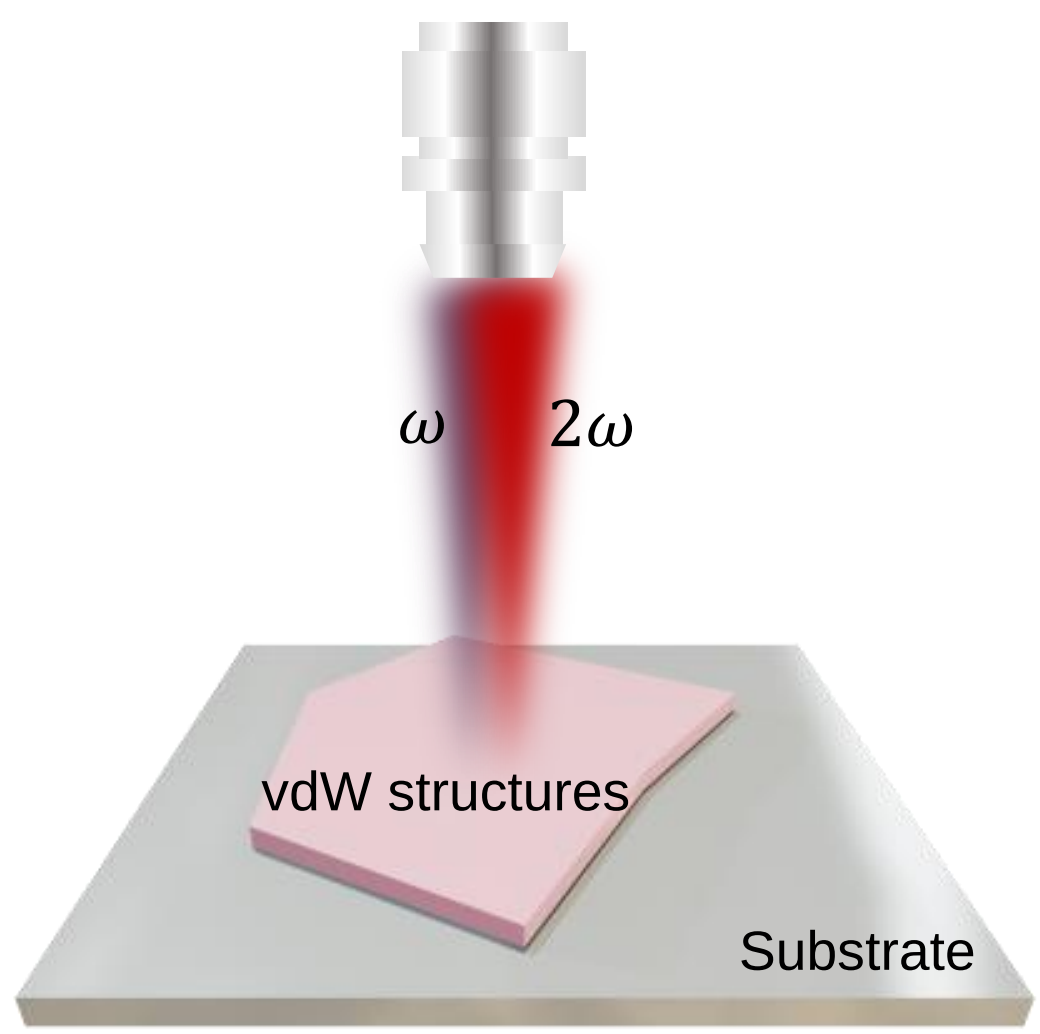

Fig. S1: **Schematic of the setup of the characterising measurement of the SH intensity with and without Al protection.**

For SHG measurements, femtosecond pulses at 800 nm wavelength and 750 kHz repetition rate are used as the excitation source, second harmonic signals, centred around 400nm, are collected in a reflection configuration, and normalised to the SHG intensity of the corresponding bare flake. The characterisation setup is schematised in Fig. S1. In the sample without an Al protective layer, the SHG intensity is substantially degraded. In contrast, the protected sample shows a notably stronger SHG response.

III. COUPLING TECHNIQUE OF MICROCAVITIES

WGMs can be excited through various coupling methods, including waveguides, tapered fibres, prisms, focused Gaussian light beams in free space, etc. Here, we use a U-shaped tapered fibre for cavity mode coupling. The preparation of tapered optical fibres involves carefully removing the outer cladding from a standard single-mode optical fibre, followed by elongation under a hydrogen flame. This process results in the formation of a double-cone

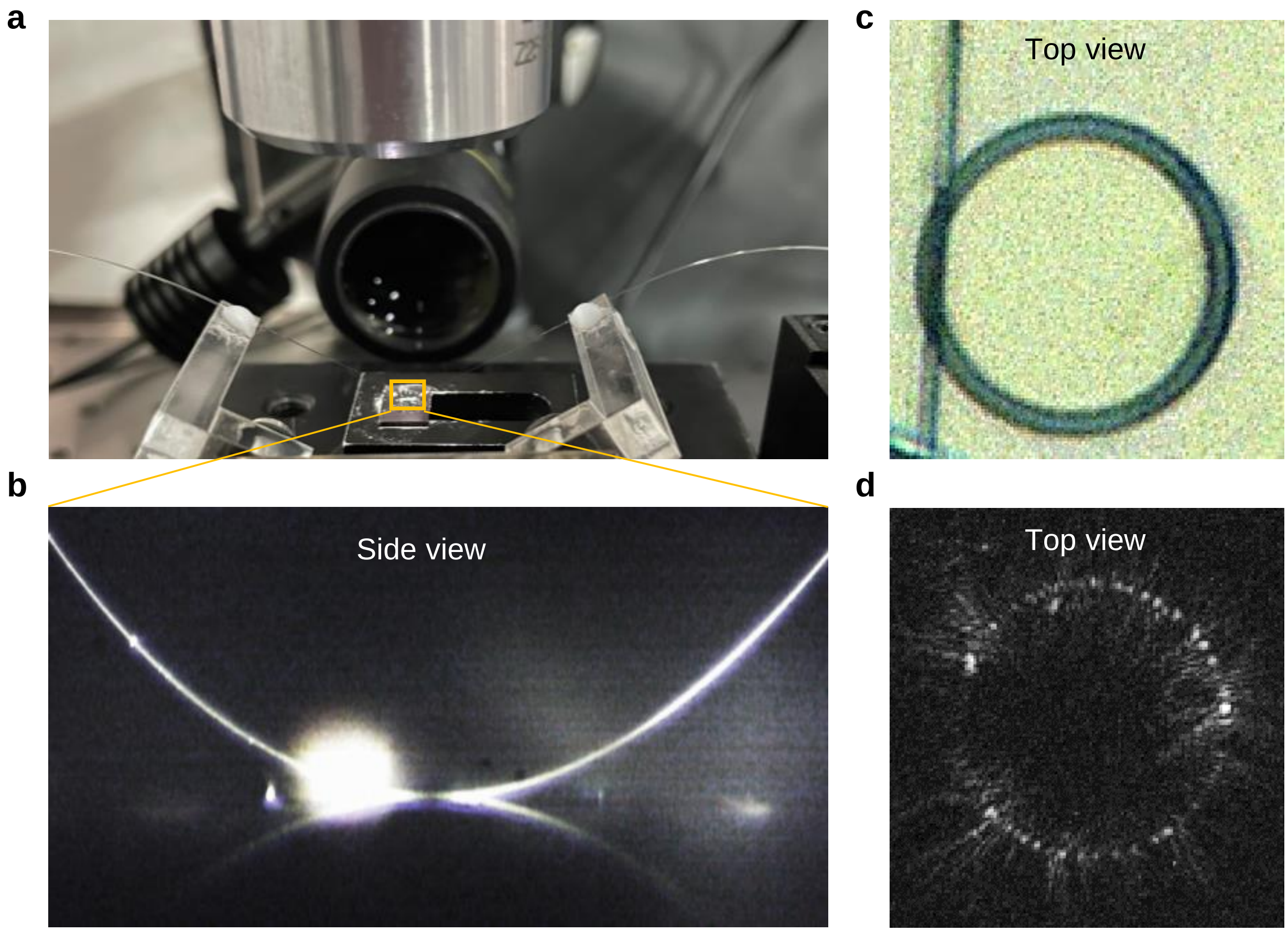

Fig. S2: **Microdisk coupled with a U-type tapered fibre. a,** Image of the coupling setup. **b,** Side view of the microdisk and coupling fibre. **c,** Top view of the microdisk and coupling fibre. **d,** Top-view dark-field optical image of the microdisk under WGM excitation.

structure in the heated region of the fibre, ensuring a smooth taper. This fibre then experiences a slight bending, and the thinnest section is then employed for evanescent coupling to the microcavity, as shown in Fig. S2**a**. WGMs are excited when both the momentum matching and the frequency matching between the fibre mode and the cavity mode are satisfied. Both top-view and side-view imaging systems are built to guarantee the right coupling

position. The pictures of the side view and top view are shown in Figs. S2**b** to **d**, exhibiting the relative position of the fibre and the cavity. In the side view, two bright curves exist, one is the real fibre image, and the other is the mirror image from the silica substrate.

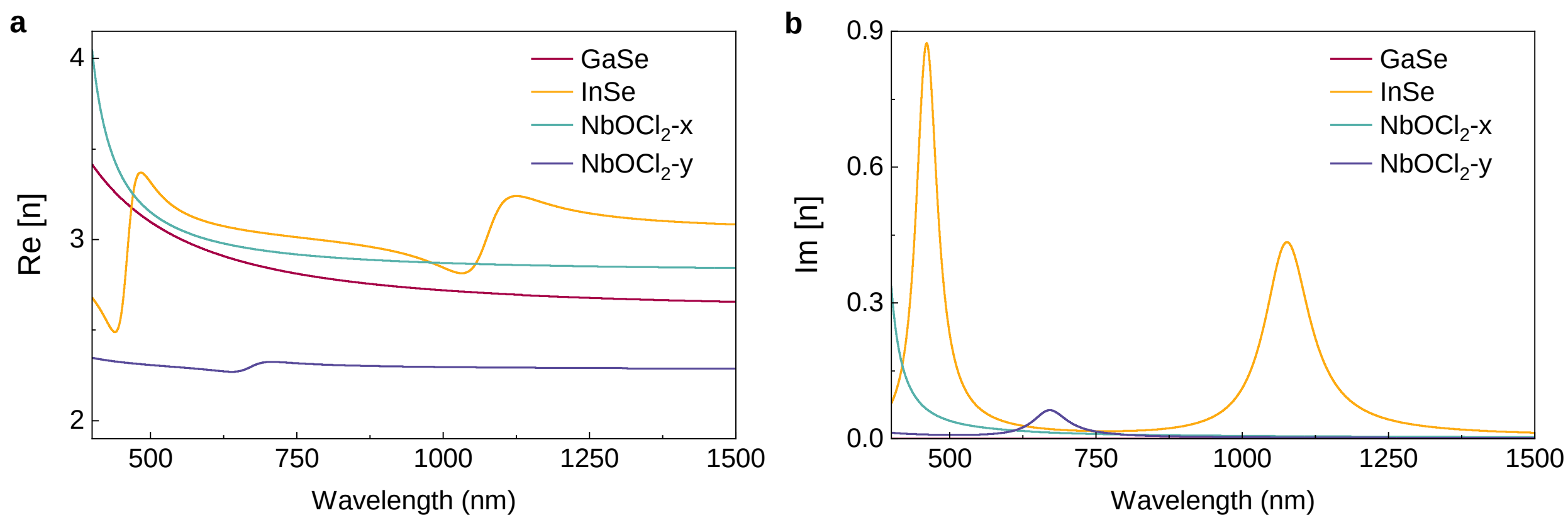

Fig. S3: **Refractive index measurement for vdW materials. a** and **b,** Real and imaginary parts of the measured refractive index of different vdW materials.

In experiments, WGMs within some vdW materials are hard to excite at some wavelength bands, due to the large momentum mismatch between the fibre and cavity with a high refractive index. Especially, the existence of the absorption peaks from the exciton process leads to a sharp change in the refractive index, hindering the excitation of WGMs and the nonlinear frequency conversion processes. Thus, the waveguide-cavity coupling requires further exploration. As a complement for further engineering for the broadband phase matching conditions of the waveguide-cavity coupling and the phase matching conditions for the nonlinear processes, we measure both the real and imaginary parts of the refractive index of several bulk vdW materials, including GaSe, InSe, and $NbOCl_2$, by angular resolved white light reflection spectroscopy [25]. The results are plotted in Figs. S3**a** and **b**, and some exciton properties disappear due to the thickness of the samples.

IV. CALIBRATION OF Q FACTORS VIA FIBRE-BASED MACH-ZEHNDER INTERFEROMETER (MZI)

In order for the measurement of the Q factor, a tunable CW light is scanned in frequency around the microcavity mode to obtain the Lorentzian-shaped transmission spectrum. The linewidth κ can be directly extracted from this spectrum, and the corresponding quality factor is calculated as $Q = \omega/\kappa$. However, the frequency-scan speed of the laser is not perfectly stable during the measurement, particularly for high-Q modes, resulting in linewidth errors. To correct for these fluctuations, a calibrated MZI is employed to precisely recalibrate the laser frequency by referencing the periodic sine-function transmission of the MZI. This calibration ensures accurate determination of the linewidth by counting the number of interference periods, as shown by the gray curve in Fig. S4**b**.

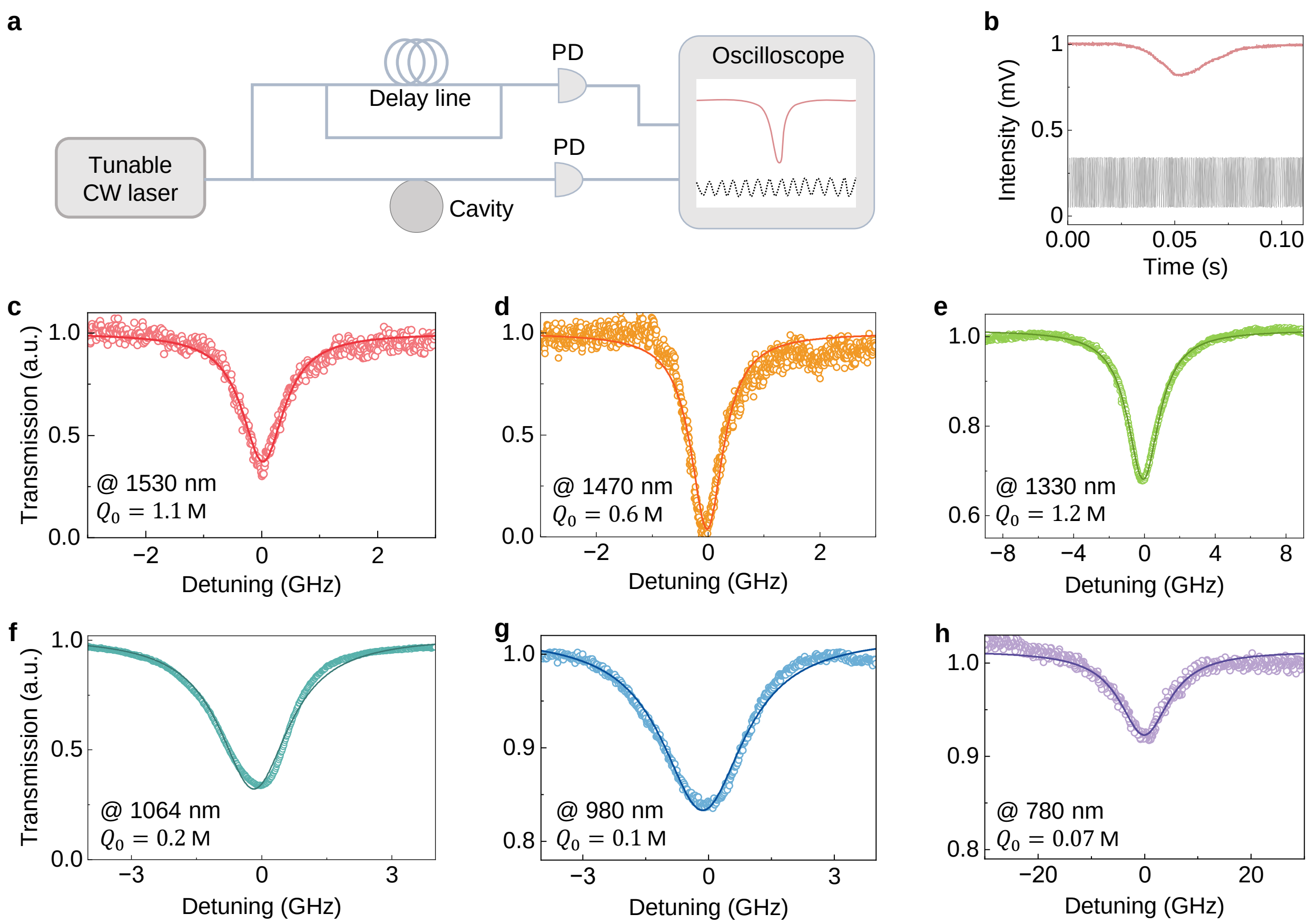

Fig. S4: **Fibre-based MZI for the calibration of Q factors. a,** Set up of the method of characterising Q factors. **b,** Signal intensity of transmissions in **a**. **c** to **h,** Transmission spectra of h-BN microcavities in different wavelength bands.

For the other thing, the total loss contains two parts: (1) the intrinsic loss κ_0, including scattering loss, absorption loss, and radiation loss; and (2) the coupling loss κ_{ex}, attributed

to the fibre coupling. The normalised transmission T at the resonant frequency can be described as

$$T = \left(\frac{\kappa_0 - \kappa_{\mathrm{ex}}}{\kappa_0 + \kappa_{\mathrm{ex}}}\right)^2. \tag{S1}$$

Therefore, the intrinsic loss and coupling loss can be extracted through the transmission. In this context, the transmission spectra of the modes in different wavelength bands are measured, and the corresponding Q factors are extracted, as exhibited in Figs. S4**c** to **h**.

Notably, the extracted linewidths for intrinsic and external ones are indistinct due to the exchange symmetry in Eq. S1. Typically, the coupling condition, which describes the relative magnitude of intrinsic loss and external coupling rate, can be determined by analysing the variation of coupling efficiency and loaded Q within a mode family. As shown in Figs. S5**a** and **c**, at 1330 nm and 1550 nm wavelength bands, the fundamental transverse electric mode family is extracted, where the loaded Q factors decrease with increasing transmission

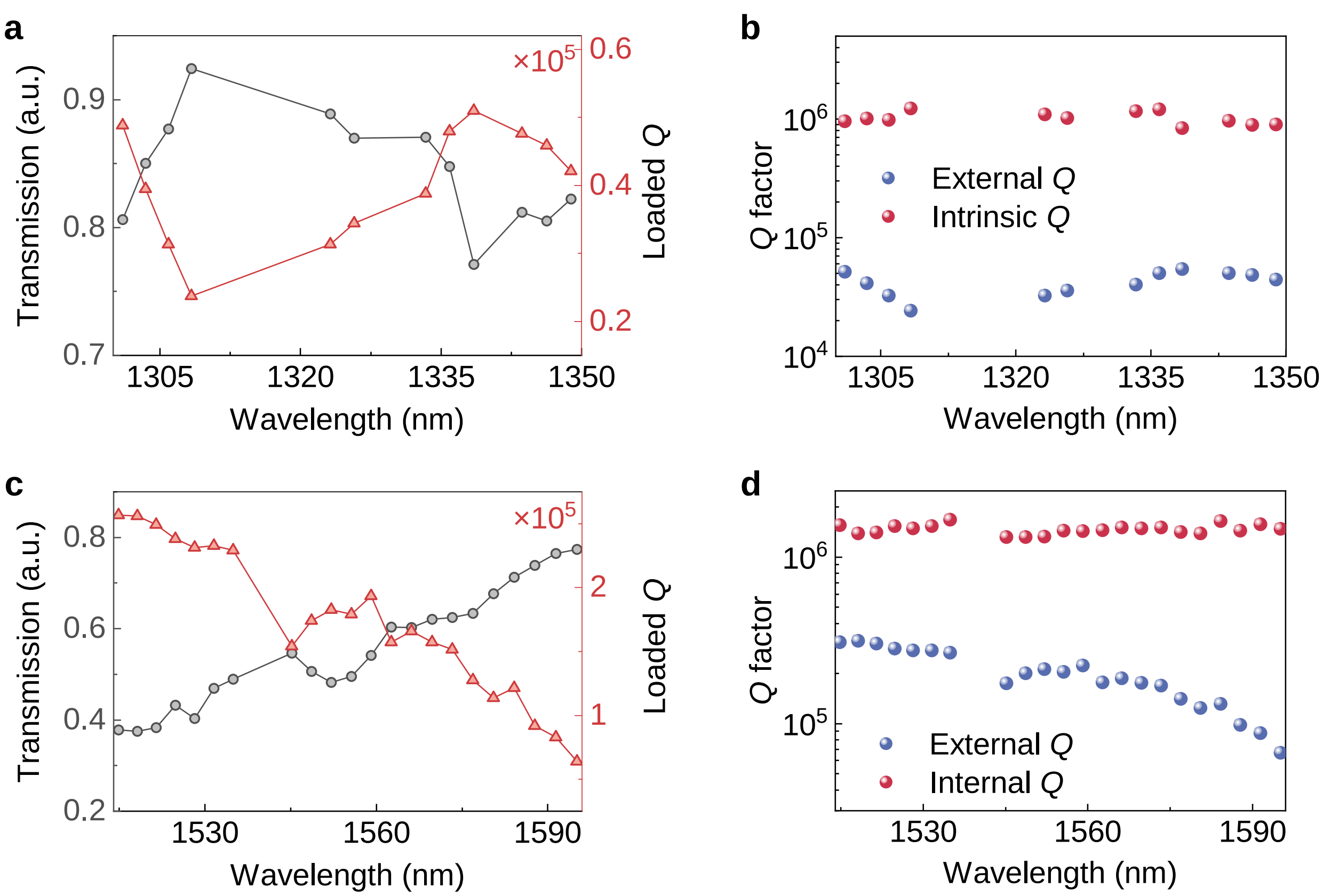

Fig. S5: **Coupling conditions at 1330 nm and 1550 nm wavelength bands. a** and **c,** Dependence of the transmission efficiency and loaded Q on the wavelength at 1330 nm and 1550 nm bands, respectively. **b** and **d,** Extracted intrinsic and external Q factors versus the wavelength at 1330 nm and 1550 nm bands.

and increase when transmission decreases. It indicates that with increasing wavelength, the external losses are increasing and the coupling efficiency is decreasing, exhibiting an over-coupling condition (i.e., $\kappa_{\mathrm{ex}} > \kappa_0$). In this context, the intrinsic and external Q factors are extracted and exhibited in Figs. S5**b** and **d**.

V. THEORETICAL MODEL OF SCATTERING LOSS

In the experiments, the Q factors of the h-BN cavity exhibit a decline as the wavelength decreases, as shown in Fig. 2d. This trend is primarily attributed to surface roughness at the cavity edge, which induces scattering loss. Such scattering loss can be modeled as dipole radiation originating from surface imperfections polarized by the local electric field [26]. The corresponding equivalent polarization current density $\vec{J}(\vec{r})$ is expressed as

$$\vec{J}(\vec{r}) = -i\omega\epsilon_0 \left(n(\vec{r})^2 - n_0^2\right) \vec{E}(\vec{r}), \tag{S2}$$

where ω is the angular frequency of light, ϵ_0 denotes the vacuum permittivity, $n(\vec{r})$ is the refractive index profile of the cavity, n_0 is the refractive index of the cladding, and $\vec{E}(\vec{r})$ is the local electric field. In the far field, the magnetic vector potential $\vec{A}$ takes the form

$$\vec{A} = \frac{\mu_0}{4\pi}\left(\frac{e^{in_0k_0r}}{r}\right)\iiint \vec{J}(\vec{r'})e^{-in_0k_0\hat{r}\cdot\vec{r'}}\mathrm{d}V', \tag{S3}$$

where k_0 is the free-space wavenumber, and μ_0 is the free space permeability. $\mathrm{d}V'$ can be written as $h(\vec{r'})\mathrm{d}S$, representing the volume element contributed by the surface imperfections.

The statistical property of the surface roughness $h(r)$ is characterized by its autocorrelation function:

$$G(u) = \langle h(r)h(r+u)\rangle. \tag{S4}$$

In most practical scenarios, this autocorrelation function can be well approximated by an exponential form:

$$G(u) = \sigma^2 \exp\left(-\frac{|u|}{L_c}\right), \tag{S5}$$

where σ^2 denotes the variance of the surface roughness, and L_c is the correlation length. The spectral density of the roughness is obtained by taking the Fourier transform of the autocorrelation function:

$$\widetilde{G}(\xi) = \int_{-\infty}^{+\infty} G(u)\exp(i\xi u)\mathrm{d}u. \tag{S6}$$

The far-field Poynting vector then simplifies to

$$\vec{S} = \hat{r}\frac{\omega n_0 k_0}{2\mu_0}|\hat{r}\times\vec{A}|^2, \tag{S7}$$

and the total radiated power is given by

$$P = \oiint \vec{S}\cdot\hat{r}\mathrm{d}S. \tag{S8}$$

Assuming the material is isotropic and applying suitable approximations, the resulting scattering power P_{sc} can be expressed as [27]

$$P_{\text{sc}} = \frac{1}{3}\sigma^2 L_c \epsilon_0^2 \mu_0 \omega^3 n_0 k_0 R (n^2 - n_0^2)^2 \cdot \left| \int_{-d/2}^{d/2} E(z) \mathrm{d}z \right|^2, \tag{S9}$$

where d is the vertical thickness of the cavity, and R is the radius of the cavity.

For anisotropic materials, however, both the refractive index distribution and the resulting electric field distribution need to be taken into account. Specifically, the refractive index and the electric field become functions of the azimuthal angle θ. Accordingly, the scattering power density along θ can be expressed as

$$\alpha_{\text{sc}}(\theta) = \frac{1}{6\pi}\sigma^2 L_c \epsilon_0^2 \mu_0 \omega^3 n_0 k_0 R (n(\theta)^2 - n_0^2)^2 \cdot \left| \int_{-\mathrm{d}/2}^{\mathrm{d}/2} E(z,\theta) \mathrm{d}z \right|^2, \tag{S10}$$

and the total scattering power is then obtained by integrating over θ:

$$P_{\text{sc}} = \int_0^{2\pi} \frac{1}{6\pi}\sigma^2 L_c \epsilon_0^2 \mu_0 \omega^3 n_0 k_0 R (n(\theta)^2 - n_0^2)^2 \cdot \left| \int_{-\mathrm{d}/2}^{\mathrm{d}/2} E(z,\theta) \mathrm{d}z \right|^2 d\theta. \tag{S11}$$

The corresponding quality factor limited by scattering loss is then given by

$$Q_{\text{sc}} = \omega \frac{W}{P_{\text{sc}}}, \tag{S12}$$

where W is the total energy stored within the cavity.

For h-BN, it is a kind of birefringent material. The optical axis is oriented perpendicular to the disk plane in our samples, so both the refractive index and the electric field remain uniform along the azimuthal angle of the cavities. Therefore, the scattering loss of h-BN cavities can be treated as the isotropic model.

This result indicates that the scattering loss is approximately inversely proportional to the cube of the wavelength. Based on this scattering loss model, the surface roughness parameters can be extracted by fitting the experimentally measured quality factors as a function of wavelength. Through fitting with experimental data, the combined parameter $\sigma^2 L_c$ of the h-BN cavity can be estimated to be around 8^3 nm^3.

VI. COMPARISON OF THE SHG EFFICIENCY AMONG VDW SYSTEMS

We list a table summarising the achieved efficiency of intrinsic vdW resonance systems in the previously reported works (Table **S2**). It is found that our maximum normalised conversion efficiency of 30%/W already exceeds most reported values, and operates under continuous-wave (CW) excitation for the first time. The efficiency enhancement factor 4.2×10^4 in the table of our platform comes from our efficiency results over that of a cavity with $Finesse = 1$. Our result is achieved without relying on double resonance or strict phase matching, but instead through the combination of ultra-high-Q factors, strong second-order nonlinearity, and large interaction volumes in vdW microcavities, indicating a strong potential for further efficiency enhancement once phase-matching and resonance conditions are fully optimised.

Table S2: Comparison of SHG conversion efficiency between our results and previously reported intrinsic vdW resonance systems.

Reference	Materials	Normalised efficiency	Absolute efficiency	Efficiency enhancement	Q factor	Phase matching		Pulse or CW pump
						Mode frequency	Momentum	
[6]	MoS_2	*5×10^{-7} % W	3.4×10^{-10} @4.3 kW peak power	/	/	No	No	Pulse
[20]	$3R-MoS_2$	/	/	100	20	No	No	Pulse
[28]	WS_2	*1.0×10^{-5} % W	5.8×10^{-9} @3.56 kW peak power	98	43.6	Yes	No	Pulse
[23]	$NbOCl_2$	/	/	25	/	No	No	Pulse
[29]	$3R-MoS_2$	/	/	150	17	No	No	Pulse
This work	GaSe	30 %/W	3.7 % @172 mW	4.2×10^4	10^5	No	No	CW

We acknowledge that our current efficiency is still several orders of magnitude lower than the record values achieved in conventional well-engineered and well-fabricated platforms with the highest efficiency of 5000000%/W [30, 31]. Looking forward, the combination of ultra-high-Q resonators and large intrinsic nonlinearity in vdW materials provides a clear route to achieving conversion efficiencies that can ultimately surpass conventional $\chi^{(2)}$ systems.

Here, we also conduct an optimisation of the device geometry to compensate for material dispersion using both geometric and modal dispersion engineering to realise the phase-matching condition [32, 33], as shown in Fig. S6. As exhibited in Fig. S6**a**, a TE_{00} mode for the FW light can match a TE_{02} mode for the SH light to realise the phase matching condition with reasonable parameters. Through adjusting the width, the phase-matching condition can also be obtained, as plotted in Fig. S6**b**.

We recognise that further development is required in terms of fabrication stability, device design, and reproducibility. Nevertheless, by leveraging insights and techniques established in conventional platforms, optimisation can be accelerated, enabling vdW integrated photonics to reach and potentially exceed the fundamental performance limits of traditional second-order nonlinear optical systems.

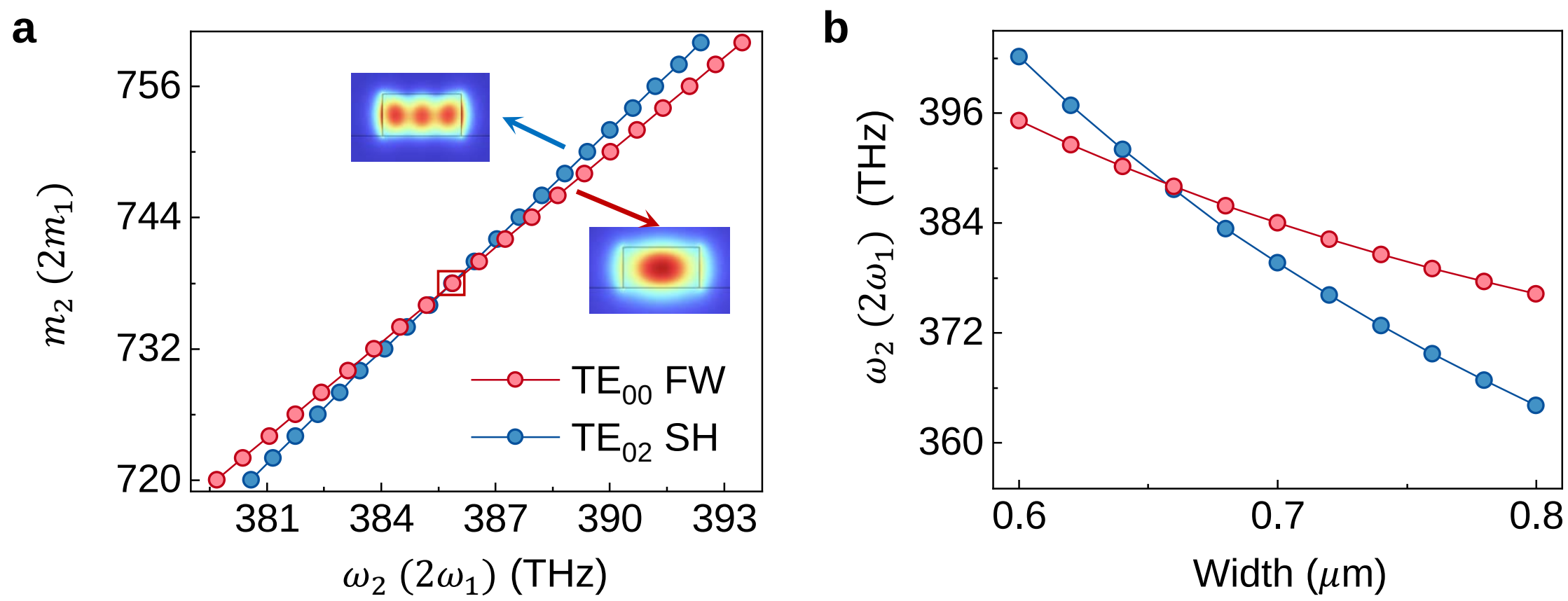

Fig. S6: **Simulation of the dispersion engineering for the phase-matching condition with ring resonance structures in the SHG process. a,** Modal dispersion engineering and **b,** Geometry dispersion engineering of the phase matching condition for the SHG process. Parameters in **a**: Width = 0.66 μm, Height =350 nm, R=43 μm. Parameters in **b**: $m_2 = 2m_1 = 744$, Height = 350 nm, R = 43 μm. $\omega_{1(2)}$ denotes the FW (SH) mode frequency and $m_{1(2)}$ represents the angular momentum number of the FW (SH) mode. Insets: Field distribution of the TE_{00} FW mode and the TE_{02} SH mode.

VII. SHG EFFICIENCY CALIBRATION METHOD

The SH light is collected through a vertical microscopic imaging system, and a highly sensitive power meter is utilised to measure the generated SH light on the image plane. In order to calibrate the overall collected efficiency of our measurement system, we inject light at the SH wavelength into the cavity. With the cavity illuminated, we put the power meter on the same image plane, and the overall collected efficiency is obtained by the ratio of the power measured at the image plane to the launched power. Specifically, for an input power of $\sim$ 205.9 µW, the collected power is $\sim$36.5 nW, yielding a overall collected efficiency of $\sim 1.77\times10^{-4}$. This collected process is totally linear, and thus, we normalise all SH power measured by the power meter by this overall collected efficiency.

VIII. DAMAGE THRESHOLD OF GASE AND THE LIMIT OF THE SHG EFFICIENCY

The damage threshold is indeed an important property, especially for some applications. We measured the damage threshold of GaSe microdisks to be ∼23 GW/cm^2 under pulsed excitation (Fig. S7). As for CW excitation, we assess the damage threshold by monitoring

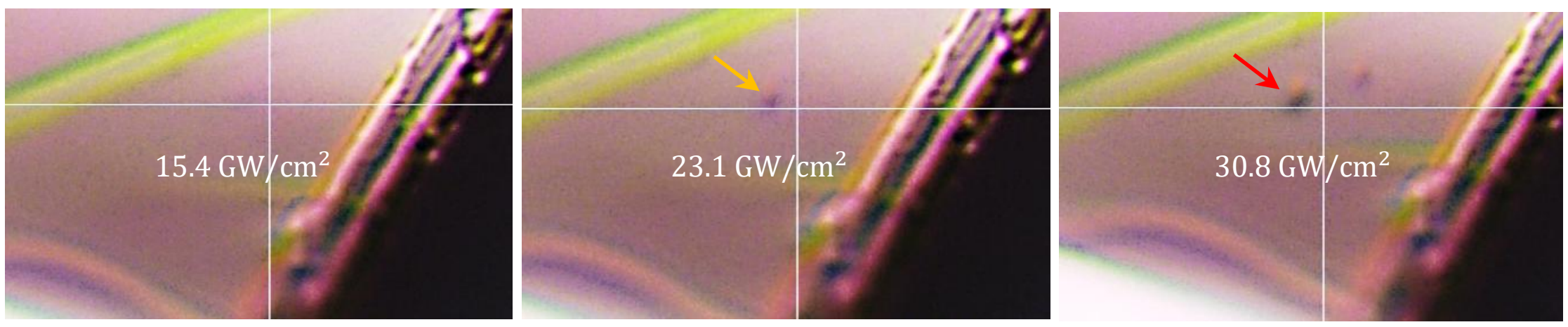

Fig. S7: **Measurement of the damage threshold of GaSe.** The thickness of the GaSe flake is 1 μm. The repetition rate of the excited pulse laser is ∼80 MHz, and the centre wavelength is 800 nm. Small damage spots (indicated by the orange arrow in the middle panel) appear at ∼23.1 GW/cm^2, giving a GaSe damage threshold of ∼23 GW/cm^2. Increasing the intensity to ∼30.8 GW/cm^2 produces a more visible damage spot (indicated by the red arrow in the right panel).

the SHG response. Figure S8 shows the SH intensity obtained when the pump is scanned into the cavity three times, producing a thermal triangle (see Fig. 3a for details). Under a ∼130 mW pump, the peak SH intensity remains essentially unchanged across the three scans. In contrast, when the pump power is increased to ∼200 mW, the peak SH intensity decreases from scan to scan, indicating a degradation of the cavity's nonlinear response. We therefore take ∼200 mW as the CW input-power damage threshold for our GaSe microdisks.

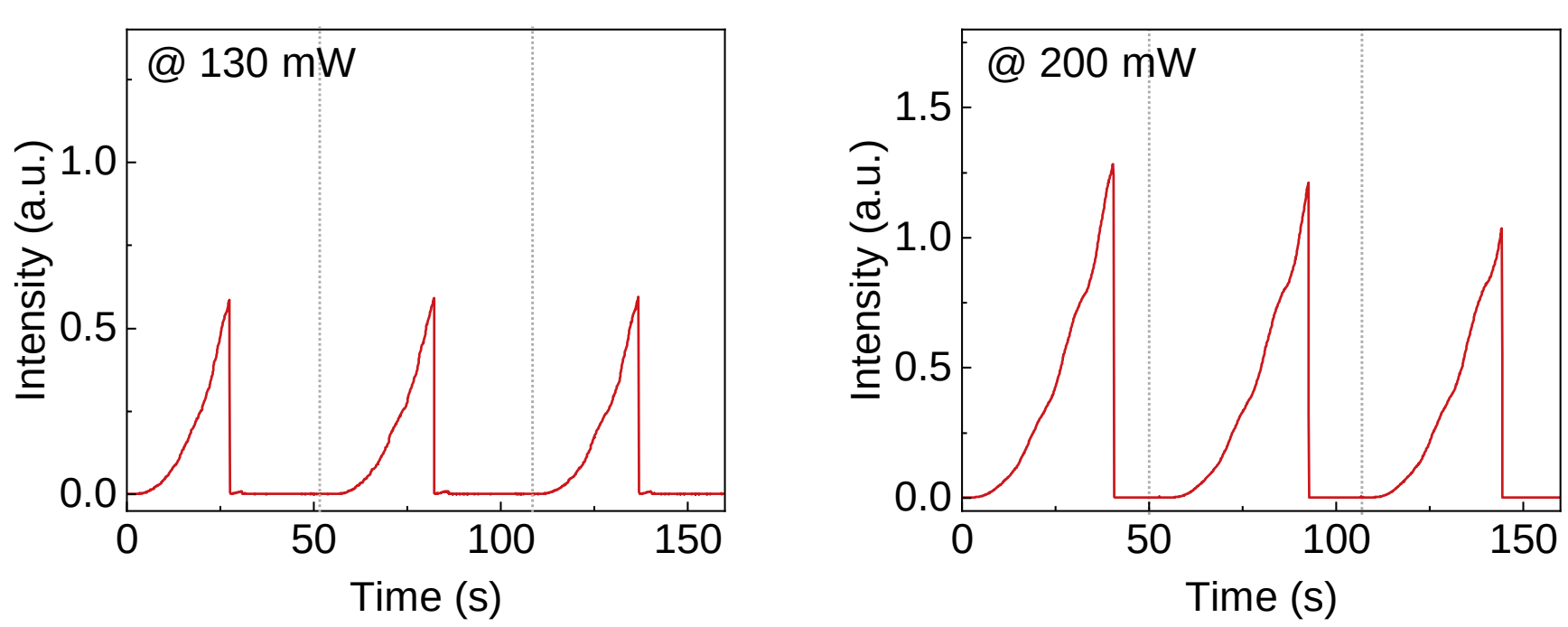

Fig. S8: **Measurements of the damage threshold under CW pump for the GaSe-microdisk device.** SH intensity versus scanning time for the pump light at different powers, with the cavity scanned three times.

IX. THERMAL MANAGEMENT FOR OUR HIGH-Q, HIGH-POWER DEVICES

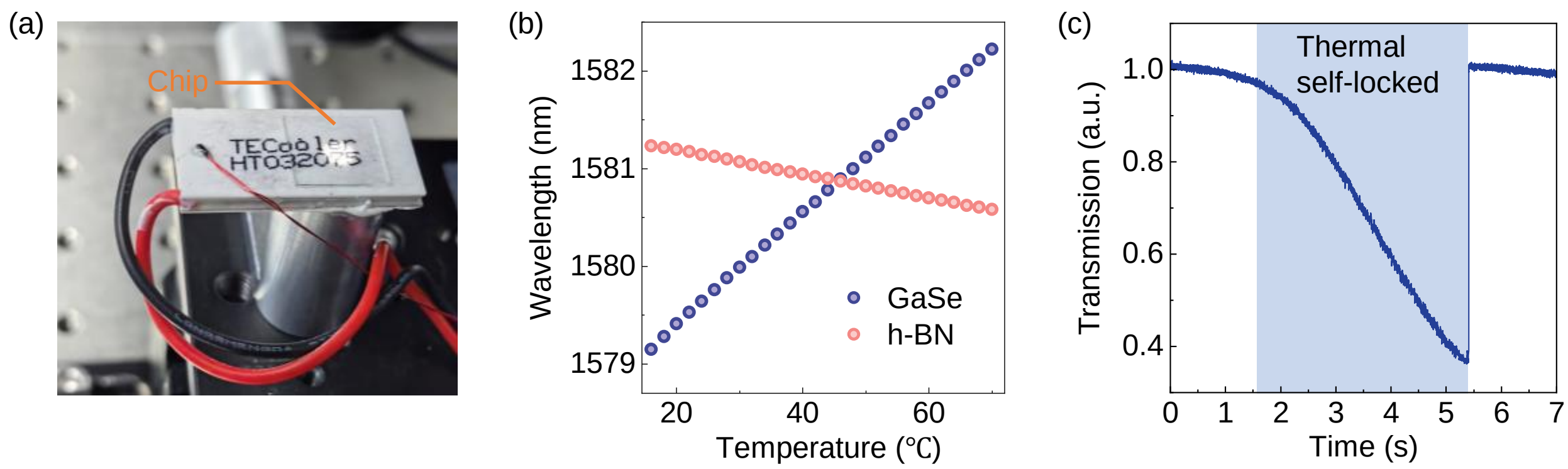

Fig. S9: **Thermal management of the device. a,** Measurement setup for temperature control and stability. **b,** Temperature dependence of the resonance wavelength for the GaSe and h-BN microdisks. **c,** Transmission spectrum of one mode under a high-power pump. The light blue shaded area depicts a thermal self-locked regime. Wavelength scanning speed: -0.02 nm/s.

The thermal management for our high-Q system is very important, especially when operating under a high-power pump. We have taken this into consideration during our measurement. As shown in Fig. S9**a**, a thermoelectric controller (TEC) is added to our chip to stabilise the substrate temperatures. This is also used to tune the resonant wavelength through temperature tuning, as presented in Fig. S9**b**. Note that, when under a high-power pump, the thermo-optic effect is obvious in modulating the mode frequency. Under certain conditions, the temperature control speed of the TEC cannot keep up with the rate of thermal fluctuations induced by the intense light within the cavity. Fortunately, there exists the thermal self-locked effect for the cavity mode, which has been fully explored in dielectric cavities [34]. Once the fall within the self-locked regime, the mode stability can be realised, ensuring robust operation even under intense optical pumping.

X. NOISE FIGURE (NF) ANALYSIS FOR THE OPA PROCESS

The noise figure is defined as

$$NF = 10 \times \log_{10} \frac{\mathrm{SNR_{in}}}{\mathrm{SNR_{out}}}, \tag{S13}$$

where $\mathrm{SNR_{in}}$ ($\mathrm{SNR_{out}}$) denotes the signal-to-noise ratio of the input (amplified) idler light. In our current implementation, due to the limited modal dispersion of the microcavity, the Kerr-OPA process itself provides only a weak signal, and correspondingly contributes little noise. As a result, the overall noise performance of the amplifier is dominated by the gain noise of the erbium-doped fibre amplifier used in our pump laser system, leading to a measured noise figure falling in the range of 3–5 dB. Looking ahead, with rigorous optimisation—particularly through dispersion engineering to enhance the amplification process and reducing reliance on the erbium-doped fibre amplifier—we anticipate that the intrinsic noise properties of Kerr-OPA can be characterised with much higher accuracy.

XI. SIDEWALL ROUGHNESS AND SURFACE EFFECT INDUCED BY AL REMOVAL

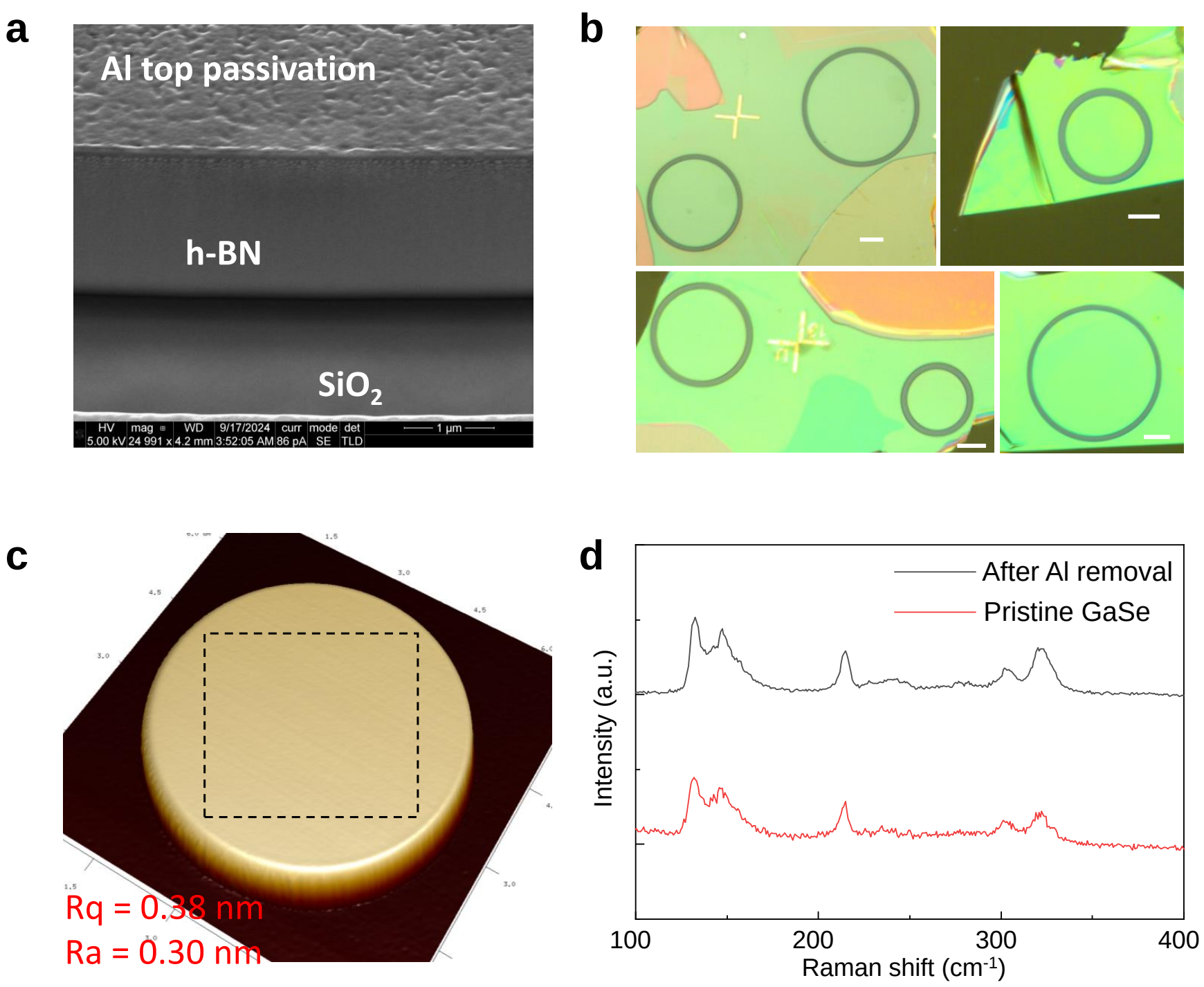

Fig. S10: **Sidewall roughness and surface effect induced by Al removal. a** The highest-magnification SEM image we took on the h-BN microdisk sample. **b** Optical microscope images that show the well-maintained GaSe flake after the wet-etching process. No visible Al residue is observed. Scale bar is 20 μm. **c** AFM characterisation of a GaSe sample after Al removal, the image is presented in 3D to have a better display of surface condition. The average roughness measured from the dashed rectangle area is 0.3 nm, indicating a well-maintained surface. **d** Raman spectra measured after Al removal, which show negligible change compared to the pristine crystal.

As shown in Fig. S10**a**, we present the highest-magnification SEM image obtained from an h-BN device for sidewall morphology characterisation. The top surface (Al passivation) is full of pits resulting from Ga-ion bombardment during pattern alignment. In contrast, the sidewall of the h-BN sample has negligible defects. Further magnification leads to image blurring due to local charges, because both h-BN and the quartz substrate are non-conductive. Further, no visible damage or residue is observed in the optical microscope images (Fig. S10**b**). Except for milled patterns, the flakes are identical to those transferred. We conducted AFM characterisation on one of the GaSe microdisks after Al removal (Fig. S10**c**). The sample surface exhibits no defect centre, and the average surface roughness is only ∼0.3 nm. We

also performed Raman analyses to confirm that the Al removal does not damage the vdW material (Fig. S10**d**). We observe no new peaks compared to pristine flakes.

Apart from the above direct morphology characterisation, the most direct and effective evidence of sidewall roughness is the high Q factor. There are mainly three sources contributing to cavity loss, including radiative loss, scattering loss, and material absorption. The substrate is quartz (n= $\sim$1.45) and the measurement is done within the transparency window (e.g., h-BN has a bandgap of $\sim$6 eV), so the dominant contribution to cavity loss is scattering from the surface. WGMs are highly sensitive to sidewall conditions, and the measured Q factor of over one million from our disks provides clear and solid evidence of sidewall smoothness.

XII. STANDARD DEVIATION OF FIB MILLING

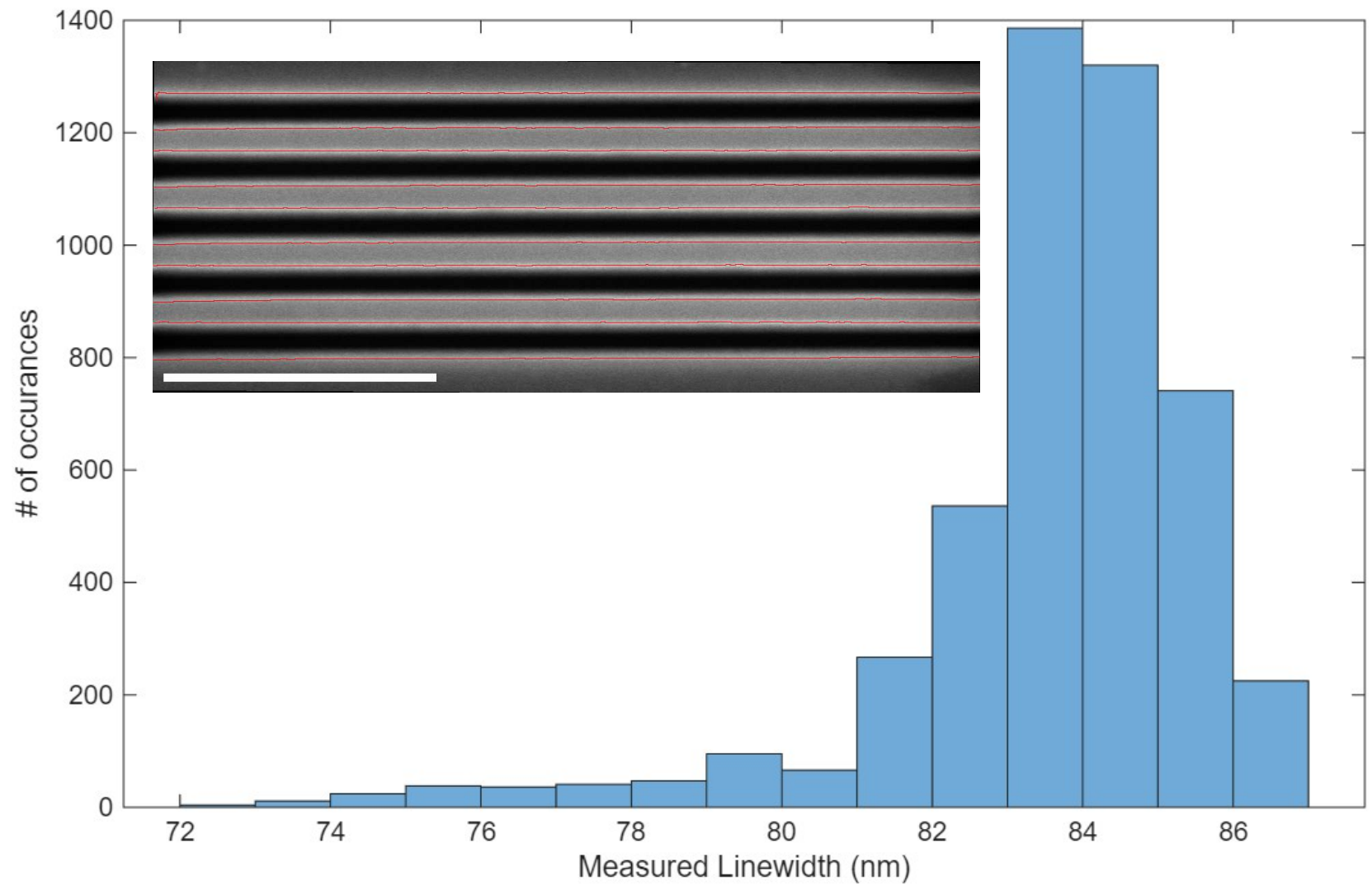

Fig. S11: **Histogram of measured linewidths.** Inset: SEM of a line-space pattern, with the outcome of the ridge detection algorithm overlayed. Scale bar: 1 μm.

To quantify the standard deviation of high-resolution structures, we analysed scanning electron micrographs of a series of closely-spaced lines (Fig. S11). To avoid measurement bias, a ridge detection algorithm was used to find the edge coordinates, from which the linewidths were calculated. The lines were found to be $\sim 83 \pm 2$ nm.

XIII. DISCUSSION ON FIB-BASED VDW MATERIAL PATTERNING

Optimisation for eliminating the wet etching process

The wet etching process in the current fabrication flow for removing the Al layer can be further optimised to extend the method to a broader range of vdW materials, such as water-sensitive black phosphorus and magnetic materials. As mentioned in the main text, the Al layer serves two roles: (1) discharging; (2) surface passivation. The discharging requirement arises from substrate selection, which aims to support our WGM-related process and application. To eliminate the discharging requirement, one could choose a conductive substrate (e.g., ITO), or fabricate the device on a conductive substrate and then transfer it to the desired non-conductive substrate. Such post-fabrication transfer has been proven feasible in previous EBL+RIE work [9]. Further, current progress on binding energy research [35] for large-scale vdW material exfoliation also provides practical routes for wet-etching-free fabrication of vdW structures through the following steps: (1) transfer a sacrificial vdW material thin layer on top of the target vdW flake, forming a heterostructure; (2) deposit the conductive discharging layer; (3) FIB programmed milling; (4) physical removal of the discharging layer via mechanical exfoliation. Well-engineered binding energy could create an adhesion difference, which only allows the transferred sacrificial vdW thin layer to be fully peeled off with the discharging layer, leaving a clean vdW surface on the patterned structure. The passivation requirement originates from the initial exposure of the flake needed for precise pattern-flake alignment. This need is tied to the tool we used, which requires a lot of manual operation and is not designed specifically for lithography. In newer dedicated FIB lithography tools that are equipped with an interferometric stage, it should be possible to align the pattern to the flake using sacrificial alignment markers, thereby eliminating the need to expose the flake to the Ga-ion beam prior to milling.

The comparison of our method to EBL+RIE-based methods

As reviewed in Table **S1** above, the RIE or ICP-RIE dry etching methods have already been widely used in fabricating vdW nanostructures, but the quality is limited, with Q factors only up to a few thousand. To achieve high-quality vdW nanostructures using RIE-based methods, there are two critical conditions needed to be satisfied: (1) the etchant gas must react with the target material, and the reaction products must be volatile (e.g., gaseous products); and (2) the mask for etching must have a high selectivity ratio and be easily removable afterwards. In addition, RIE-based dry etching requires a high-quality mask, which is typically fabricated by EBL. However, the choices of effective etchants are extremely limited. Using non-reactive etchants greatly prolongs the processing time, and making a hard mask becomes the only viable option (either metallic, such as the Al employed in our FIB process, or dielectric like SiN). The mask post-removal and maintaining good

resolution during a long-time etch process are very challenging. Moreover, some etchants react with the material and form extremely stable compounds that block further etching (e.g., Fluorine-based etchants with InSe). Others produce reaction byproducts that significantly alter material properties (e.g., Bi_2Se_3 to BiF_3). Further, even if under certain conditions when both etchant gas and mask are suitable, the use of photoresist also requires wet chemical processes such as spin-coating, development, and post-removal. The most successful examples of this type of method are 3R-MoS_2 [20] and WS_2 [3, 9], in which remarkably strong light–matter interactions were reported. Nonetheless, the best Q factors reported for these devices are only a few hundred to a few thousand.

Our method using FIB is a physical process. This eliminates many of the aforementioned constraints and offers a much simpler fabrication-flow, considering the need for mask fabrication and removal in RIE-based methods. Regarding the Q factor, our method achieves a clear three orders of magnitude improvement over previous vdW material works based on EBL+RIE. Leveraging this high Q factor, we demonstrate remarkable light–matter interaction and further showcase this enhancement through nonlinear optical processes. Therefore, our method can provide a useful toolkit and will surely benefit the whole community across many different research fields.

Scalability of our method

The FIB tool (Helios Nanolab600) used in this work is a dual-beam SEM/FIB designed primarily for the lift-out of TEM lamellae, and is not a true lithography platform. The results presented herein are limited by the need for skilled manual operation of a tool that has a limited write-field area and with only rudimentary drift compensation. Notably, true FIB lithography platforms have been produced in recent years, e.g., from Raith. These platforms enable large-area lithography due to the interferometrically-controlled stage, which allows for reproducible sample positioning and accurate stitching of write-fields. Systems like these are also inherently more stable than the tool we used in this work and can run unsupervised for many hours with automatic drift compensation. As such, there is potential for the results presented herein to be extended at least up to small-batch production.

Wafer-scale fabrication perspectives

With the fast progress in material synthesis, wafer-scale vdW materials are expected to be gradually available, promising a photonic integrated circuit fully manufactured using vdW materials. However, integrating vdW nanostructures with existing platforms is also a pathway worth investigating, particularly integrating vdW nanostructures as functional units, as most existing platforms still suffer from challenges in compatible hybrid integration for multifunctionality. Using vdW nanostructures is quite promising to fill this gap. For example, h-BN has an index of $\sim$2, which perfectly matches the Si_3N_4 platform. To facilitate such hybrid integration, one possible way is to dry transfer the fabricated vdW nanostruc-

tures to the target location. The transfer of vdW nanodisks has been proved feasible [9]. The precision of this transfer is certainly still a question, but it should have an optimistic future with further optimisation. While the transfer of individual structures is laborious and therefore not amenable to mass fabrication, using post-fabrication transfer would also enable the use of conductive substrates during FIB milling, and thereby potentially eliminate the need for an Al layer.

Beyond the fabrication technique itself, the vdW materials nanostructure we studied in this work has already shown great potential in loss performance. Expectedly, the upper limit of the loss performance of vdW materials has not yet been reached. Nevertheless, there are ample opportunities to further exploit the inherent advantages of layered vdW crystals. With the rapid progress in vdW material synthesis and fabrication techniques, large-scale, high-quality production is expected to become feasible [36], ultimately paving the way for a mature material platform that follows roadmaps similar to established ones (e.g., silicon-on-insulator, lithium niobate on insulator, silicon nitride, etc.).

[1] Kim, S. *et al.* Photonic crystal cavities from hexagonal boron nitride. *Nature Communications* **9**, 2623 (2018).

[2] Fröch, J. E., Hwang, Y., Kim, S., Aharonovich, I. & Toth, M. Photonic nanostructures from hexagonal boron nitride. *Advanced Optical Materials* **7**, 1801344 (2019).

[3] Verre, R. *et al.* Transition metal dichalcogenide nanodisks as high-index dielectric Mie nanoresonators. *Nature Nanotechnology* **14**, 679–683 (2019).

[4] Busschaert, S. *et al.* Transition metal dichalcogenide resonators for second harmonic signal enhancement. *ACS Photonics* **7**, 2482–2488 (2020).

[5] Khelifa, R. *et al.* Coupling interlayer excitons to whispering gallery modes in van der Waals heterostructures. *Nano Letters* **20**, 6155–6161 (2020).

[6] Nauman, M. *et al.* Tunable unidirectional nonlinear emission from transition-metal-dichalcogenide metasurfaces. *Nature Communications* **12**, 5597 (2021).

[7] Li, C. *et al.* Integration of hBN quantum emitters in monolithically fabricated waveguides. *ACS Photonics* **8**, 2966–2972 (2021).

[8] Li, C. *et al.* Room-temperature near-infrared excitonic lasing from mechanically exfoliated InSe microflake. *ACS Nano* **16**, 1477–1485 (2021).

[9] Sung, J. *et al.* Room-temperature continuous-wave indirect-bandgap transition lasing in an ultra-thin WS_2 disk. *Nature Photonics* **16**, 792–797 (2022).

[10] Feng, S. *et al.* Deterministic and scalable generation of exciton emitters in 2D semiconductor nanodisks. *Advanced Optical Materials* **10**, 2102702 (2022).

[11] Zotev, P. G. *et al.* Transition metal dichalcogenide dimer nanoantennas for tailored light–matter interactions. *ACS Nano* **16**, 6493–6505 (2022).

[12] Popkova, A. A. *et al.* Nonlinear exciton-Mie coupling in transition metal dichalcogenide nanoresonators. *Laser & Photonics Reviews* **16**, 2100604 (2022).

[13] Cui, X. *et al.* On-chip photonics and optoelectronics with a van der Waals material dielectric platform. *Nanoscale* **14**, 9459–9465 (2022).

[14] Vyshnevyy, A. A. *et al.* Van der Waals materials for overcoming fundamental limitations in photonic integrated circuitry. *Nano Letters* **23**, 8057–8064 (2023).

[15] Ling, H. *et al.* Deeply subwavelength integrated excitonic van der Waals nanophotonics. *Optica* **10**, 1345–1352 (2023).

[16] Lee, S. W., Lee, J. S., Choi, W. H. & Gong, S.-H. Ultrathin WS_2 polariton waveguide for efficient light guiding. *Advanced Optical Materials* **11**, 2300069 (2023).

[17] Nonahal, M. *et al.* Engineering quantum nanophotonic components from hexagonal boron

nitride. *Laser & Photonics Reviews* **17**, 2300019 (2023).

[18] Kühner, L. *et al.* High-Q nanophotonics over the full visible spectrum enabled by hexagonal boron nitride metasurfaces. *Advanced Materials* **35**, 2209688 (2023).

[19] Munkhbat, B., Küçüköz, B., Baranov, D. G., Antosiewicz, T. J. & Shegai, T. O. Nanostructured transition metal dichalcogenide multilayers for advanced nanophotonics. *Laser & Photonics Reviews* **17**, 2200057 (2023).

[20] Zograf, G. *et al.* Combining ultrahigh index with exceptional nonlinearity in resonant transition metal dichalcogenide nanodisks. *Nature Photonics* **18**, 751–757 (2024).

[21] Weber, T. *et al.* Intrinsic strong light-matter coupling with self-hybridized bound states in the continuum in van der Waals metasurfaces. *Nature Materials* **22**, 970–976 (2023).

[22] Sortino, L. *et al.* Optically addressable spin defects coupled to bound states in the continuum metasurfaces. *Nature Communications* **15**, 2008 (2024).

[23] Chu, L. *et al.* Van der Waals $NbOCl_2$ nanodisks for enhanced second-harmonic generation. *Nano Letters* **24**, 15457–15463 (2024).

[24] Osiekowicz, M. *et al.* Resonance and antiresonance in Raman scattering in GaSe and InSe crystals. *Scientific Reports* **11**, 924 (2021).

[25] Wang, T. *et al.* Magnetically-dressed CrSBr exciton-polaritons in ultrastrong coupling regime. *Nature Communications* **14**, 5966 (2023).

[26] Wu, L. *et al.* Hydroxyl ion absorption in on-chip high-Q resonators. *Optics Letters* **48**, 3511–3514 (2023).

[27] Ciminelli, C., Passaro, V. M., Dell'Olio, F. & Armenise, M. N. Three-dimensional modelling of scattering loss in InGaAsP/InP and silica-on-silicon bent waveguides. *Journal of the European Optical Society-Rapid Publications* **4**, 09015 (2009).

[28] Nauman, M. *et al.* Dynamic control of nonlinear emission by exciton-photon coupling in WS_2 metasurfaces. *Science Advances* **11**, eady2108 (2025).

[29] Ling, H. *et al.* Nonlinear van der Waals metasurfaces with resonantly enhanced light generation. *Nano Letters* **25**, 9229–9236 (2025).

[30] Lu, J., Li, M., Zou, C.-L., Al Sayem, A. & Tang, H. X. Toward 1% single-photon anharmonicity with periodically poled lithium niobate microring resonators. *Optica* **7**, 1654–1659 (2020).

[31] Akin, J., Zhao, Y., Misra, Y., Haque, A. N. & Fang, K. Ingap $\chi^{(2)}$ integrated photonics platform for broadband, ultra-efficient nonlinear conversion and entangled photon generation. *Light: Science & Applications* **13**, 290 (2024).

[32] Trovatello, C. *et al.* Quasi-phase-matched up-and down-conversion in periodically poled layered semiconductors. *Nature Photonics* **19**, 291–299 (2025).

[33] Xu, X. *et al.* Towards compact phase-matched and waveguided nonlinear optics in atomically

layered semiconductors. *Nature Photonics* **16**, 698–706 (2022).

[34] Carmon, T., Yang, L. & Vahala, K. J. Dynamical thermal behavior and thermal self-stability of microcavities. *Optics Express* **12**, 4742–4750 (2004).

[35] Moon, J.-Y. *et al.* Layer-engineered large-area exfoliation of graphene. *Science Advances* **6**, eabc6601 (2020).

[36] Liu, C. *et al.* Understanding epitaxial growth of two-dimensional materials and their homostructures. *Nature Nanotechnology* **19**, 907–918 (2024).
